# Overhauser Dynamic Nuclear Polarization of Lithiated Graphite Anodes: Probing Bulk and Surface Structures

**DOI:** 10.1021/acs.chemmater.5c00845

**Published:** 2025-07-01

**Authors:** Teresa Insinna, Anne-Laure Barra, Clare P. Grey

**Affiliations:** † Yusuf Hamied Department of Chemistry, 2152 University of Cambridge, Lensfield Road, Cambridge CB2 1EW, United Kingdom; ‡ LNCMI-CNRS, EMFL, Univ. Grenoble-Alpes, 25 Rue des Martyrs, B.P. 166, 38042 Cedex 9 Grenoble, France

## Abstract

Graphite is used, almost ubiquitously, as an anode material in today’s high energy density Li-ion batteries. Both artificial and natural graphites are widely used, and there are large differences in the production methods, cost, particle morphologies, sizes, and percentage of defects in their structures, all these parameters affecting use and performance. The success of graphite as an anode depends on the formation of a Li-ion-conducting passivation layer (the solid electrolyte interphase (SEI)) on the first cycle, with the nature of this layer still being under investigation with a range of approaches. During lithiation (charge in a full cell), graphite is lithiated in stages and becomes electronically conductive. The conduction electrons of lithiated graphite anodes are exploited in this work to enhance the nuclear magnetic resonance (NMR) signal of bulk and surface nuclei via Overhauser dynamic nuclear polarization (DNP). The parameters directly affecting the enhancement factor (leakage factor, saturation factor, and coupling factor) are examined in detail for an artificial graphite at different lithiation stages. Four additional (natural and artificial) graphites are then studied to explore the effects of particle size and morphology, electron relaxation times, and conductivity on the observed DNP enhancements. Finally, the polarization transfer between bulk and surface (SEI) species is explored through ^6,7^Li, ^1^H, and ^13^C DNP NMR experiments.

## Graphical abstract

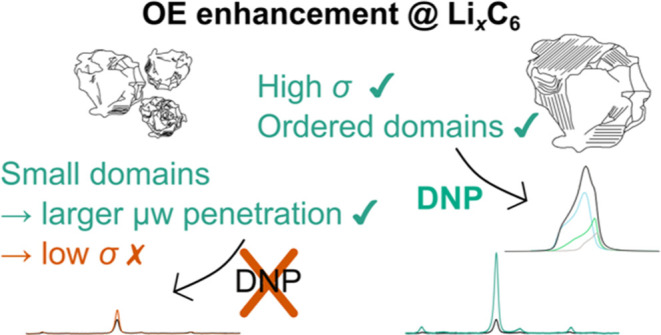

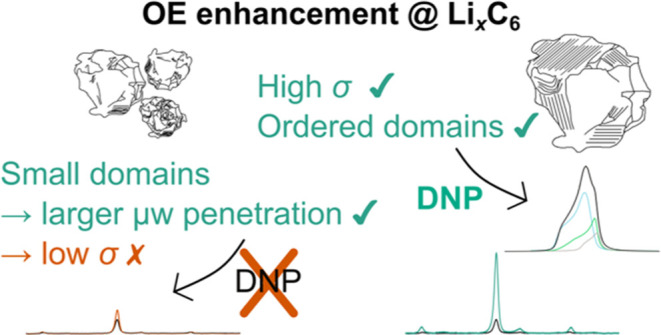

## Introduction

Graphite anodes are the most successful anode materials for lithium-ion batteries (LIBs) to date,^1,2^ with high reversible capacities and long lifetimes. Despite their success, graphite anodes are still subject to a series of degradation reactions that occur during battery cycling. Of particular importance is the Solid Electrolyte Interphase (SEI), a passivating layer comprised of both organic and inorganic components, formed by electrochemical reduction and decomposition of the electrolyte at the surface of the graphite electrode,^3–5^ which protects the electrolyte from further reduction while also allowing Li^+^ ions to pass through it. The nature of the SEI is heterogeneous: its composition and thickness vary depending on the applied currents/potentials, the nature of the electrolyte and any additives, and crossover of any degradation products from the positive electrode. The extent of passivation through the SEI is also strongly dependent on particle size and morphology.^5,6^

The graphites used in LIBs can be broadly divided into two groups: artificial and natural. Artificial graphites are typically made by heating carbon precursors (e.g., coke) up to 2500 °C.^7,8^ Natural graphites are instead obtained through mining and usually subjected to additional treatments such as pitch coating to reduce their surface areas; this decreases the extent of Li^+^ lost to SEI formation on the intercalated graphite, reducing capacity loss.^9^ Artificial graphites, although more expensive (currently US$13/kg vs US$8/kg for natural graphites),^10^ tend to have better performance and longer lifetime, thanks to their lower specific surface area.^10^

The composition of the SEI on graphite anodes has been extensively studied with a variety of different techniques, including X-ray photoelectron spectroscopy (XPS),^5^ titration,^11^ solution and solid state nuclear magnetic resonance (NMR) spectroscopy,^12,13^ and single ion mass spectrometry (SIMS).^14^ While depth profiling can, to a certain degree, be performed by using XPS and SIMS, the inherent roughness of the surfaces and the materials themselves introduces considerable inaccuracy. Further, these techniques can be destructive, owing to the low vacuum pressures applied and high-energy ionizing radiation used. Solid state NMR, with its atomic specificity, is often preferable as a nondestructive tool with which to probe the local structure of this amorphous and highly disordered layer.^15,16^ The lack of sensitivity of traditional solid state NMR can then, in principle, be enhanced through dynamic nuclear polarization (DNP) NMR methods, which can be highly surface specific depending on the approach used.^17,18^

Critical to understanding the SEI is the determination of the phase(s) at the SEI–Li metal and SEI–graphite interphases, since these phases help to prevent further reduction of the electrolyte, while also allowing Li-ion transport across the interphase. While most techniques lack selectivity to the anode–SEI interface, this was achieved recently in Li metal anodes, where the Li–SEI interface was probed by transferring polarization from the Li metal conduction electrons via an Overhauser Dynamic Nuclear Polarization (OEDNP) process.^19^ This DNP mechanism is activated by endogenous radicals (those inherent to the material), such as the unpaired electrons present at the Fermi level of lithium metal, rather than exogenous radicals (those added to the system)—as were, for example, added in the study of the SEI formed on reduced graphene oxide (r-GO)^20^ to enhance the solid state NMR signal of the SEI components.

Overhauser DNP relies on a hyperfine-coupled electron–nuclear system, where saturation of the EPR single-quantum transition by microwave irradiation is accompanied by cross-relaxation events (see below) to produce hyperpolarization of the coupled nuclear spins (see Figure 1). For the cross-relaxation events to occur, motion or fluctuations (i.e., spectral density) must be present at or close to the frequencies of the zero or double quantum transitions (ZQ/DQ) of the coupled spin system (as shown in Figure 1). In Li metal, the mobility of the intrinsic conduction electrons satisfies this condition.^21,22^ Given that graphite becomes metallic (it is a semimetal in the pristine state) upon intercalation of Li ions (Li_*x*_C_6_, with 0 < *x* < 1, as reported by others and us),^23–26^ we hypothesized that the Overhauser mechanism could be exploited in this system too.

**Figure 1:**
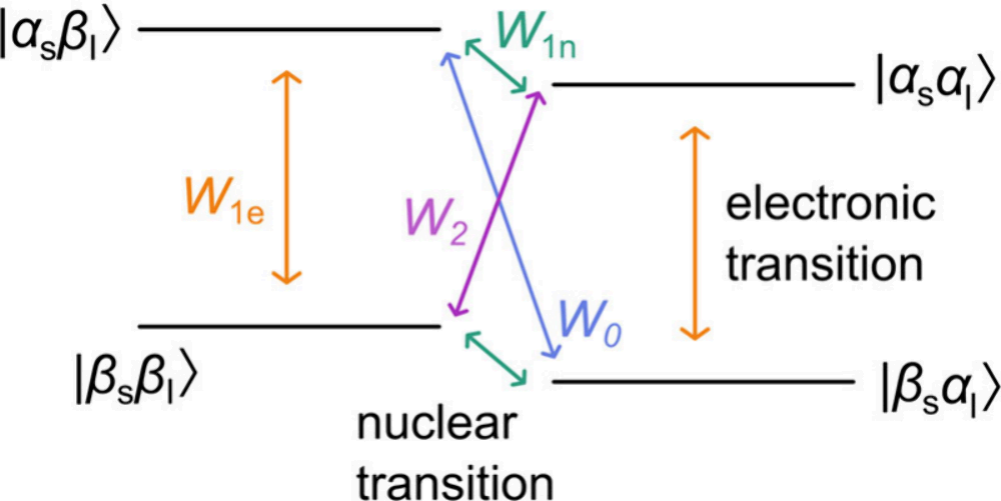
Energy level diagram of a one electron (denoted by *S*)–one nucleus (denoted by *I*) system and the transitions that can occur in Overhauser DNP, with α and β denoting spin up and spin down configurations. *W*_1e_ is the rate constant of the EPR transition, with Larmor frequency ω_1e_, *W*_1n_ is the rate constant of the NMR transition, ω_1n_, and *W*_2_ and *W*_0_ describe the rate constants of the double quantum and zero quantum cross-relaxation transitions.

Unlike Li metal, where the conduction band is dominated by Li-based states, there are two different overlapping bands that contribute to the density of states at the Fermi level in lithiated graphite, formed from the Li 2*s* and C 2*p*_*z*_ electrons. Furthermore, while Li has a cubic crystal structure, lithiated graphites have hexagonal, anisotropic crystal structures in all the various stages, with consequences for their (anisotropic) electronic properties and conductivity.^27,28^ The conduction electrons in lithiated graphite are delocalized over the C layers, interacting with Li ions between the layers via hyperfine coupling, as shown by our EPR^24^ and previous NMR studies^23^ and studies of its electronic structure more broadly.^29^ Therefore, signal enhancement via Overhauser DNP could occur for ^6,7^Li and ^13^C within the bulk as well as for any nuclei (such as ^6,7^Li, ^13^C, and ^1^H) present in surface (SEI) species generated during battery cycling.

Here we report the first example of high-field Overhauser DNP as applied to lithiated graphite anodes. First, a very brief introduction to Overhauser DNP in metals is provided; then we explore in detail the factors affecting the Overhauser mechanism in the context of an artificial graphite anode (from Hitachi), whose EPR properties we previously reported on;^24^ finally, we apply this methodology to a range of artificial and natural graphites. In doing so, we correlate particle size and morphology with EPR properties, electronic structure, and Overhauser DNP efficiency.

An overview of the Overhauser effect is given with more detail in the SI, although the reader is directed to refs 21 and 30–34 for a more in-depth discussion of the physics behind it. Briefly, we consider a coupled two-spin system comprising an electron *S* and a nuclear spin *I* with energy levels defined by *m*_s_ and *m*_I_ and separated by the respective Larmor frequencies. Transitions between the electronic states rely on microwave irradiation at the (allowed) EPR transition frequency, while transitions between nuclear states rely on rf irradiation at the corresponding nuclear Larmor frequencies. When continuous microwave irradiation is applied at a frequency corresponding to the EPR transition, electron spins are excited to their excited states. At this stage, different processes can occur (Figure 1): the spins can relax down to the ground states via single quantum processes (with rate *W*_1e_) as quantified by the electron spin–lattice relaxation time, *T*_1e_ (1/*W*_1e_). The electron spins can also interact with the nuclear spins via the hyperfine interaction, inducing spin flips of both the electron and nuclear spins. Both double quantum (DQ, with rate *W*_*2*_) cross-relaxation, where |*αα*⟩ → |*ββ*⟩, and zero quantum (ZQ, *W*_*0*_) cross-relaxation, |*αβ*⟩ → |*βα*⟩ can occur, the relative contributions depending on the nature of the hyperfine interaction, as discussed later. The enhancement depends on the relative rates of these relaxation processes, along with other experimental factors. These can all be captured in the following equation (with the derivation outlined in more detail in the SI):^30^

1 ε=⟨Iz⟩I0=1−ξfs|γe|γI

where ⟨*I*_*z*_⟩ is the nuclear magnetization in the *z*-direction, *I*_0_ the nuclear equilibrium magnetization, ξ the coupling factor, *f* the leakage factor, *s* the saturation factor, and *γ*_e_ and *γ*_I_ the electron and nuclear gyromagnetic ratios. These factors will be discussed below in the context of our lithiated graphites.

## Materials and Methods

### Materials

Hitachi graphite electrodes were fabricated in the Argonne National Laboratory Cell Analysis, Modeling and Prototyping (CAMP) facility from a mixture of 92.0 wt % graphite powder (Hitachi MagE3), 2.0 wt % carbon black (Timcal C45), 6.0 wt % polyvinylidene fluoride (PVDF) binder (Kureha 9300), and 0.17 wt % oxalic acid; this mixture was coated on Cu foil. The areal loading of the graphite active material was 5.83 mg cm^–2^. Lifun (artificial) graphite electrodes were sourced through Lifun (Kaijin AML-400); they were fabricated from a mixture of 95.0 wt % graphite, 1.2 wt % carbon black, and 3.8 wt % water-soluble binder; they had an areal loading of 11.3 mg cm^–2^. The Timrex KS44 graphite was purchased from Timcal; SPG graphite was obtained from a source in China; and the BM graphite was obtained by ball milling Timrex KS44 powder for 60 min in a planetary ball mill. The Timrex, BM, and SPG electrodes were made by mixing 90 wt % of the respective graphite powder with 5 wt % Super Porous Carbon (SuperP Li, Timcal) and 5 wt % PVDF binder (Kynar homopolymer) with *N*-methyl-2-pyrrolidone (NMP) into a slurry, which was then coated on Cu foil (300 μm wet thickness). The graphite electrodes were punched out into discs (12.7 mm diameter), dried under vacuum at 120 °C for 12 h, and then transferred to an Ar glovebox without exposure to air. LP57 electrolyte (1.0 M LiPF_6_ in ethylene carbonate, EC:ethylmethyl carbonate, EMC, 3:7 v/v, battery grade, Sigma-Aldrich or Solvionic) was used. Li metal discs were purchased from LTS Research Laboratories, Inc. Glass microfiber (Whatman GF/B) separators, cut into 16 mm disks, were used. All procedures described below (coin cell assembly and disassembly, EPR capillary preparation, and rotor packing) were performed in an Ar-filled glovebox with water and oxygen levels below 5 ppm.

### Electrochemistry

All electrochemical tests were performed in graphite/Li half-cells in 2032 coin cells (Cambridge Energy Solutions). One graphite electrode disc, a glass fiber separator soaked with 100 μL of LP57 electrolyte, and one Li metal disc were stacked and assembled into the coin cell.

The cells were cycled galvanostatically (constant-current cycling, CC) at room temperature (20 ± 2 °C) on a VMP2 potentiostat (Biologic) at a rate of *C*/23 based on a cell capacity of 360 mAh g^–1^). All potentials reported in this work are referenced against Li/Li^+^. The graphite samples studied here were lithiated in half cells once to a voltage cutoff of 0.001 V for dense stage 1, 0.076 V for stage 2 and 0.12 V for stage 2L.

### X-band Continuous-Wave EPR Spectroscopy

*Ex situ* cw EPR spectroscopy was carried out on an X-band benchtop EPR spectrometer (E5000, Magnettech) set at a microwave frequency of 9.477 GHz. The samples were loosely packed into low-background 1 mm o.d. glass capillaries and (where noted) in 1.3 mm o.d. zirconia NMR rotors. A modulation field of 0.1 mT was applied at a 100 kHz modulation frequency. Except where otherwise stated, a microwave power of 1 mW was applied. The *g*-factor was calibrated using a Mn^2+^ in ZnS standard.

### High-Frequency cw EPR Spectroscopy

High-frequency (HF) cw EPR spectra were recorded on a double-pass transmission EPR spectrometer at the Laboratoire National des Champs Magnétiques Intenses (LNCMI, Grenoble, France).^35^ The frequency was set to 331.2 GHz using a 110.4 GHz frequency source associated with a tripler, while detection was achieved using a bolometer. Temperatures were monitored using a variable-temperature insert (Cryogenic). The spectra were collected at 50 K at a sweep rate of 0.2 mT s^–1^, using a modulation field of 0.84 mT at a 0.7 kHz modulation frequency. The graphite powders were packed in 4 mm (outer diameter) Quartz tubes (Wilmad, Sigma-Aldrich) and the powders were covered in nonane (99%, anhydrous, Sigma-Aldrich) to prevent them from undergoing torquing effects. The tubes were sealed by using epoxy glue in an Ar-filled glovebox. Phase correction of the HFEPR spectra was achieved through an in-house processing script using spherical harmonics, as described elsewhere.^24^ The EPR spectra were fitted to a powder pattern line-shape with anisotropic *g* using the EasySpin toolbox for MATLAB.^36^ The fitted *g*-tensors were then corrected by using a field calibration factor obtained from a control sample (Mn^2+^ in MgO).^37^ A phase fitting parameter in Easyspin was used to account for phasing caused by the skin depth. EasySpin was also used to simulate the absorption EPR spectrum using the same fitting parameters as for stage 1 in ref. 24 at 100 K and assuming a microwave frequency of 263 GHz (at 9.4 T).

### Scanning Electron Microscopy

SEM images were acquired on pristine powder graphite samples with a Tescan MIRA3 FEG-SEM instrument at an acceleration voltage of 5.0 kV.

### Dynamic Nuclear Polarization (DNP)

The majority of the DNP measurements were carried out on a 9.4 T Bruker Avance Neo DNP spectrometer equipped with a 264 GHz klystron microwave source and a power supply unit (Lakeshore Inc.) to sweep the field. The experiments were carried out in a wide-bore low temperature magic angle spinning (LTMAS) 1.3 mm probe at different temperatures (as specified in the main text). The temperature was calibrated using the nuclear spin–lattice, *T*_1_, relaxation time of ^79^Br in KBr for the temperature range 100–250 K and using the temperature, *T*, dependence of the ^79^Br shift at *T* > 250 K. Turning on microwaves (klystron) increased the sample temperature by ∼1–2 K at *T* < 250 K and by ∼2–3 K at 280–300 K. Additional experiments were performed at the Centre de Resonance Magnétique Nucléaire (CRMN) à Très Haut Champs in Lyon, France, on a 9.4 T Bruker Avance DNP spectrometer equipped with a 264 GHz gyrotron microwave source and a power supply unit (Lakeshore Inc.) to sweep the field, using a 1.3 mm probe and a sample temperature of 170 K. The temperature change using the gyrotron was not quantified with KBr, but a more significant sample heating is expected, since the emitted power is higher than for the klystron.

All samples were packed under Ar from cycled electrodes into 1.3 mm ZrO_2_ outer diameter rotors. The powders were diluted with powdered quartz by mass, as specified in the main text. The MAS frequency was 20 kHz, with spinning performed under N_2_, unless specified otherwise.

^7^Li spectra were recorded using a rotor-synchronized Hahn echo sequence (90° – τ – 180° – τ – acquire) or a one-pulse sequence (90° – acquire); no significant change (less than approximately 10%) in the signal intensity was observed between spectra acquired using either one pulse or Hahn echo sequences when using a klystron microwave source, hence the Hahn echo was preferred due to its cleaner baseline; when using a gyrotron, a one-pulse sequence was preferred since the higher microwave power from the gyrotron was expected to induce larger heating effects and potentially result in signal loss (from shortening of spin–spin *T*_2_ relaxation times) in the echo. Saturation recovery measurements were performed with and without microwaves (klystron and gyrotron) over a range of temperatures. Very little change in the intercalated Li *T*_1_ times was observed (e.g. *T*_1,OFF_ = 2.0 s and *T*_1,ON_ = 1.8 s at 290 K using a klystron; *T*_1,OFF_ = 4.1 s and *T*_1,ON_ = 3.9 s at 170 K using a gyrotron).

The ^7^Li radiofrequency (RF) pulses used an 83 kHz field strength, the pulse length being calibrated on the graphite samples due to their metallicity affecting the nutation frequency. Briefly, in metals longer pulses are required to flip the magnetization by 90° due to the rf strength being attenuated by skin effects.^38^ The ^7^Li chemical shift was referenced to solid LiF as an external reference (−1.00 ppm). ^1^H spectra were acquired using a rotor-synchronized Hahn echo pulse sequence, with rf pulses with a 166 kHz field strength, the pulse length and the chemical shift being referenced to glycine (8.00 ppm at 20 kHz MAS)^39^ or adamantane (1.90 ppm). Cross-polarization experiments were calibrated on the sample, with the carrier frequencies set at the frequencies corresponding to the center of the ^1^H resonance and ^7^Li SEI peaks for ^1^H and ^7^Li, respectively.

## Results

### SEM Analysis and Electrochemistry of the Graphites Studied in This Work

DNP studies were performed on five types of graphite, to probe whether different intrinsic particle sizes, degrees of disorder and conductivity played a role in the efficiency of the DNP mechanism. These are the Hitachi MagE3, Timrex KS44, both commercial graphites, the ball milled version of the latter (henceforth named BM), a commercial Lifun graphite, and a SPG (spherodized) graphite sample. They are all artificial graphites, except for the SPG graphite, which is of natural origin.

The SEM images of the five graphites can be seen in Figure 2. The average particle size was measured using ImageJ software over 70+ particles of each of the graphites yielding an average of 13 μm for the Hitachi graphite, 10 μm for the Timrex graphite, 7 μm for Lifun, 9 μm for the SPG graphite, and 6 μm for the BM graphite (the full details are given in Table 1). The Hitachi, Timrex and Lifun graphites have a flake-like morphology (Figure 2 (a-c)), with the first two appearing as agglomerates of smaller particles (hence the wider particle size distribution); the BM graphite maintains the flakelike morphology but with a smaller average particle size and higher level of disorder (the particles appear as fragments, Figure 2(e)). The SPG (Figure 2(d)) has a spheroid-like morphology as is usually the case for natural graphites that have been spheridized and then coated with pitch to smooth the surface and fill any pores.^40^

**Figure 2:**
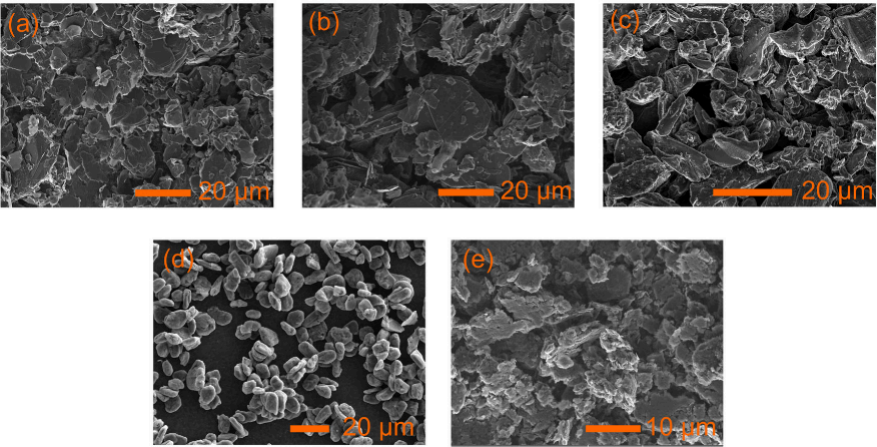
SEM images of the five different graphites examined in this study: (a) Hitachi MagE3, (b) Timrex KS44, (c) Lifun, (d) SPG, and (e) Ball milled Timrex (BM).

**Table 1:** Table Summarizing Properties of the Five Different Graphites Examined Here (Hitachi MagE3, Timrex KS44, Lifun, SPG, and Ball Milled Timrex)^a^

Graphite type	Average size (μm)	Size distribution (min–max in μm)	ε_LiC6_	ε_SEI_	Δ*B*_pp_ (mT)	A/B
Hitachi	13 ± 12	2–77	4	3	0.20	2.29
Timrex	10 ± 9	1–46	4	3	0.22	1.91
Lifun	7 ± 5	1–20	3	2	0.24	1.96
SPG	9 ± 2	4–13	1.5	4	0.51	1.73
Ball milled	6 ± 3	1–17	2.3^a^	1.4^a^	0.92	1.19

a The properties are average particle diameter, size distribution, enhancements of the LiC_6_ and the SEI resonances, X-band EPR peak-to-peak line width, and asymmetry parameter. Errors in particle size are 1 SD of the average as determined by averaging over 70+ particles with ImageJ software. Enhancements are the ratio of the intensity of the ON/OFF spectra.

b Recorded at 280 K using a klystron microwave source, while the other enhancements were recorded at 170 K using a gyrotron microwave source.

The five graphites were lithiated to a composition close to LiC_6_ corresponding to stage 1 (cutoff voltage, 1 mV vs Li/Li^+^, Figure 3), and the electrodes were extracted for the DNP measurements. The very large capacities observed for the BM sample and to a lesser extent the Lifun and Timrex carbons are mainly due to electrolyte degradation on the graphite’s surface (at 0.9 V for BM, 0.7 V for Hitachi, Timrex and SPG and ∼0.5 V for Lifun). Separate samples containing predomantly stage 2, LiC_12_, and dilute stage 2L (composition close to LiC_31_) were also prepared using the Hitachi graphite electrodes by stopping at different cutoff voltages (see Methods for further details and Figure S2 for electrochemistry).

**Figure 3:**
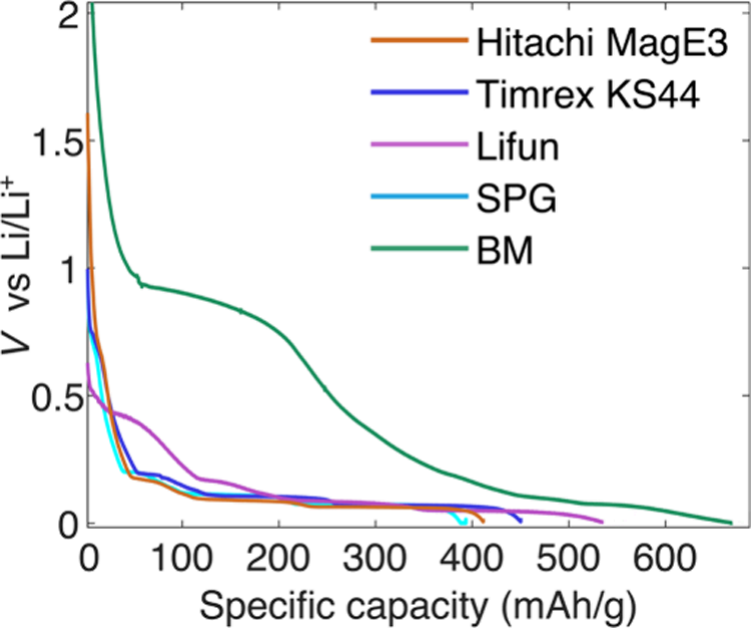
Voltage profile acquired under constant current for the first lithiation of the five different graphites studied in this work. The graphite electrodes were discharged against Li metal with LP57 electrolyte at a rate of C/23 (assuming a specific capacity of 360 mAh/g) until a voltage cutoff of 1 mV vs Li/Li^+^.

### OEDNP in Lithiated Graphite Anodes

#### DNP Field Sweep Profile

To confirm whether any endogenous DNP could be performed on lithiated graphite anodes and to determine the mechanism, a ^7^Li sweep profile was first conducted over a 0.018 T field range (∼2000 ppm) on a 9.4 T DNP spectrometer equipped with a klystron continuous wave (cw) microwave source on a sample of LiC_6_ prepared with Hitachi graphite (for electrochemistry see Figure 3). The LiC_6_ sample was extracted from the electrochemical cell and diluted by grinding with quartz powder (90 wt %) in an Ar glovebox and packed into a 1.3 mm rotor. Quartz was chosen as a diluent since it did not react with the lithiated graphites, at least on the time scale of these measurements. The ^7^Li MAS NMR spectrum of this sample was dominated by a peak at 42.6 ppm consistent with stage 1 graphite (LiC_6_) (Figure 4(a)).

**Figure 4:**
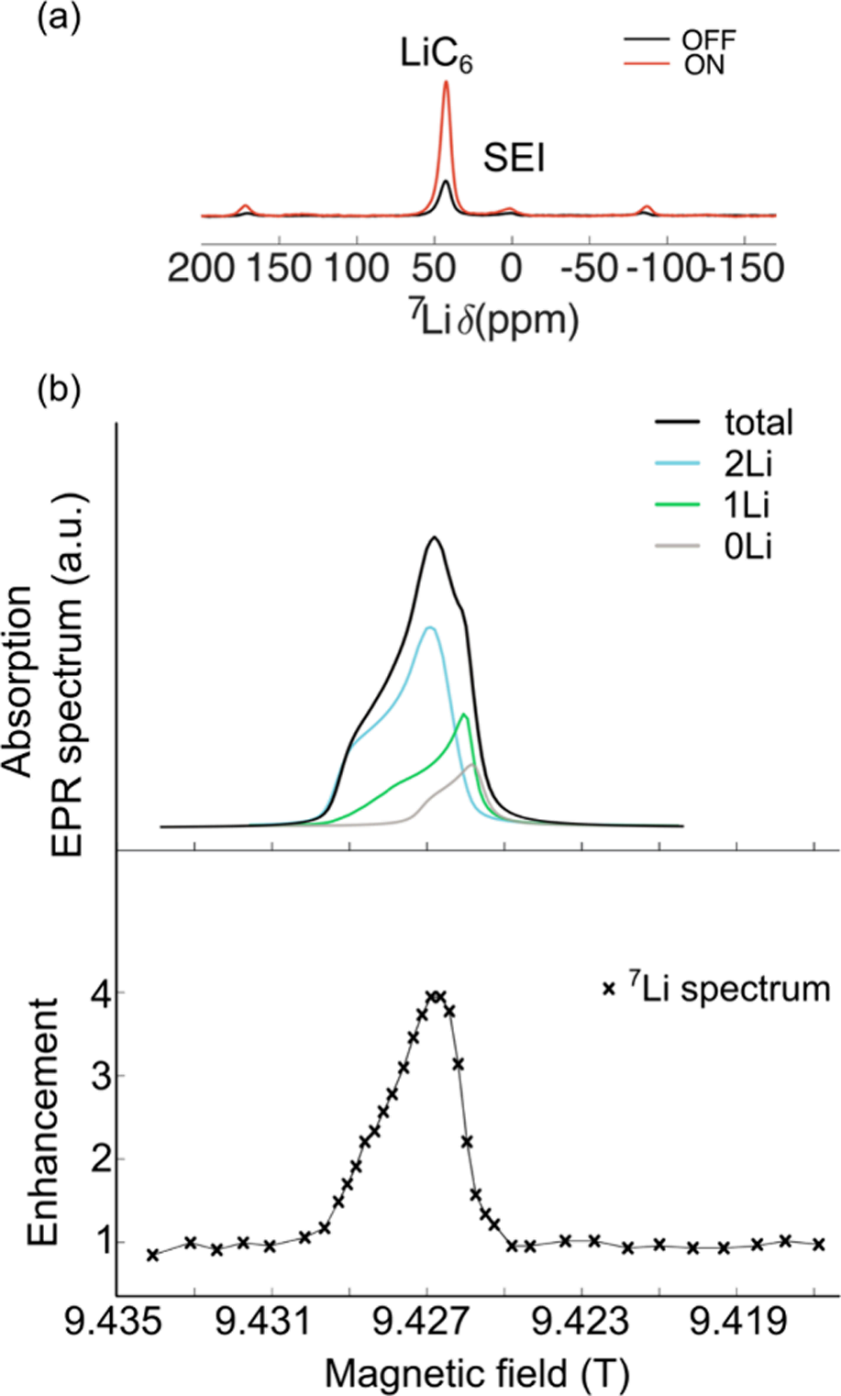
(a) ^7^Li ON/OFF spectra of LiC_6_ prepared from Hitachi graphite and diluted with quartz (90 wt % quartz) at 250 K. The shifts of the two isotropic resonances are given; the remaining peaks are spinning sidebands. The spectra were acquired with a MAS frequency of 20 kHz and a recycle delay of 5 s. (b) DNP field sweep profile of LiC_6_ at 9.42 T and 250 K (bottom). The field profile shows only one maximum confirming that the mechanism at play is the Overhauser effect, with the maximum enhancement being observed at the maximum of the conduction electrons’ EPR line. The EPR line shape, simulated as an absorption curve using the same parameters extracted from the fit of the LiC_6_ acquired at 331 GHz as reported in our previous work (ref 24) is shown for comparison at the top. The fitting parameters are reported in Table 2.

A DNP field sweep performed at 250 K revealed only one enhancement maximum at a frequency corresponding to the EPR transition of fully lithiated graphite (LiC_6_; Figure 4(b), with the ON/OFF ^7^Li MAS NMR spectra at the maximum shown in Figure 4(a)). The enhancement profile is relatively sharp, but broader than the Li metal field profile,^19,41^ consistently with the broader EPR line shape of LiC_6_ (∼0.2 mT) vs. that of Li dendrites (∼0.01–0.001 mT, depending on the Li morphology).^42^ The profile is not, however, symmetric, with the lower field side rising faster to the maximum than the higher field side, which decays more slowly. This is attributed to the presence of *g*-anisotropy in the electronic environments of LiC_6_, which can be resolved at high field.^24^ A simulation of the absorptive EPR spectrum using the same *g*-factor, *g*-anisotropy, hyperfine coupling and line widths as previously extracted from the LiC_6_ EPR spectrum in ref.^24^ produces a very similar profile to the field sweep (Figure 4(b), top).

A positive ^7^Li enhancement of the LiC_6_ resonance (42.6 ppm) of ∼4.0 is observed, which is smaller than that typically observed for Li metal,^19^ the positive enhancement being consistent with the presence of a Fermi contact interaction between the mobile conduction electrons in lithiated graphite and the Li ions, resulting in unpaired electron density at the nuclear position. In addition to the intercalated Li ions, the surface Li-containing species (within the SEI, ∼0 ppm) are also enhanced with an enhancement of ∼2.5. As mentioned in the introduction, the enhancement can be affected by several factors (eq 1) that are now explored.

#### Leakage Factor

The leakage factor quantifies the signal loss caused by the paramagnetic relaxation enhancement (PRE)—*i.e.*, relaxation determined by the proximity (via Fermi contact and/or dipolar hyperfine interactions) of the unpaired/conduction electron to the observed nucleus. In systems with extrinsic radicals (e.g., as used for Overhauser DNP in liquids, for exogenous DNP, or for metal-ion DNP), the effect of the PRE can be quantified by taking the ratio of the spin–lattice *T*_1_ relaxation time in the presence (*T*_1,*rad*_) and absence (*T*_1,*norad*_) of the radical source:^43^

2 $$f=1-\frac{T_{1,rad}}{T_{1,norad}}$$

In a metal, this is quite challenging, as the radical source is intrinsic to the material. To overcome this issue, two approaches were taken. On one hand, the leakage factor can be described in terms of rates of nuclear relaxation occurring via the hyperfine interaction (*R*_1_,_*hyp*_) vs. the total of the relaxation rates occurring via all possible relaxation mechanisms (e.g., by dynamics, etc.) (*R*_1,*total*_)^30^

3 f=W0+2W1e+W2W0+2W1e+W2+W1n=R1,hypR1,total=T1,totalT1,hyp

In this case, if the *T*_1_ is solely due to metallic relaxation, then the leakage factor is 1. Metallic relaxation in (simple) metals obeys the Korringa relation, i.e. *T*_1_ × *T (temperature in K)* = constant.^44^ It has been shown that for the dense stages of graphite (LiC_6_ and LiC_12_) the ^7^Li *T*_1_ relaxation follows this Korringa behavior, while in the dilute stages relaxation is also strongly influenced by dynamics of the Li ions within the layers.^23^ Therefore, at least for the dense stages, the leakage factor *f* should be close to 1.

An alternative approach to estimate the leakage factor is to exploit the observation that the metallicity of graphite is tuned by controlling the degree of lithiation.^23,24,27,45^ Since dilute stages of graphite are only semiconducting/semimetals and not metallic, their measured *T*_1_ times can be used as estimates of *T*_1,*norad*_. The *T*_1_ values of the metallic phases LiC_6_ and LiC_12_ are similar (1.69 and 2.03 s respectively at room temperature) while in the dilute stage 2L (LiC_31_) it is noticeably longer (8.20 s).^23^ Using these values and eq 2, leakage factors of *f*_LiC6_ = 0.79 and *f*_LiC12_ = 0.75 are calculated. These estimates can only be considered as a lower bound for *f* since the *T*_1_ time for LiC_31_ contains a contribution from relaxation due to motion of the Li^+^ ions that is not present in the dense stages and also likely contributions to relaxation from their semiconducting/semimetal electronic structure.^46^ Since both methods produce a leakage factor close to 1, this suggests that at least for the dense stages, the enhancement from the OEDNP is not limited (primarily) by this term.

#### Saturation Factor

The saturation factor is defined as

4 $$s=\frac{\left(S_{0}-\left\langle S_{z}\right\rangle \right)}{S_{0}}=\frac{\omega_{1}^{2}T_{1e}T_{2e}}{1+\omega_{1}^{2}T_{1e}T_{2e}}$$

where ω_1_ = *γB*_1_ is the Rabi frequency of the electron spins at the microwave field *B*_1_, which can be thought of as a proxy for the microwave power.^47^ The saturation factor therefore identifies the ability of microwave irradiation to saturate the EPR transition (of the conduction electrons). It is dependent on the microwave power, with higher power corresponding initially to an EPR signal increase and, on saturation, to a progressive signal drop. The ability to saturate the EPR transition depends also on the *T*_1e_ (and *T*_2e_) of the electrons, with shorter *T*_1e_ resulting in a short-lived excited state and therefore a lower chance of successful cross-relaxation events. In metals, it is often assumed that *T*_1e_ = *T*_2e_,^48^ although other factors such as the skin (and spin depth) may affect measurements of these paramaters.^24^

The *T*_1e_s of four different stages of lithiated (Hitachi) graphite were previously measured using X-band EPR (*B*_0_ = 0.336 mT for *g* = 2) and are reproduced here in Figure 5(b);^24^ the *T*_1e_s were not temperature independent (as reported for Li metal),^49^ but rather they increased to reach a maximum before dropping (in the dense stages) or gradually decreasing (for the dilute stages) as the temperature was lowered. The presence of antiferromagnetic interactions—likely arising from defects or edge effects—were, at least in part, thought to account for the decreases at low temperatures.^24^ We note that the temperature independence of the *T*_1e_ of lithium metal, with its small spin–orbit coupling and thus relatively long electronic relaxation times, is the exception. While for Li metal relaxation is dominated by impurity scattering, in most other metals the relaxation mechanisms are controlled by a coupling between the motions of the electrons and the vibrations of the lattice, and thus they decrease with temperature, resulting in a lengthening of the *T*_1e_s.^49^

**Figure 5:**
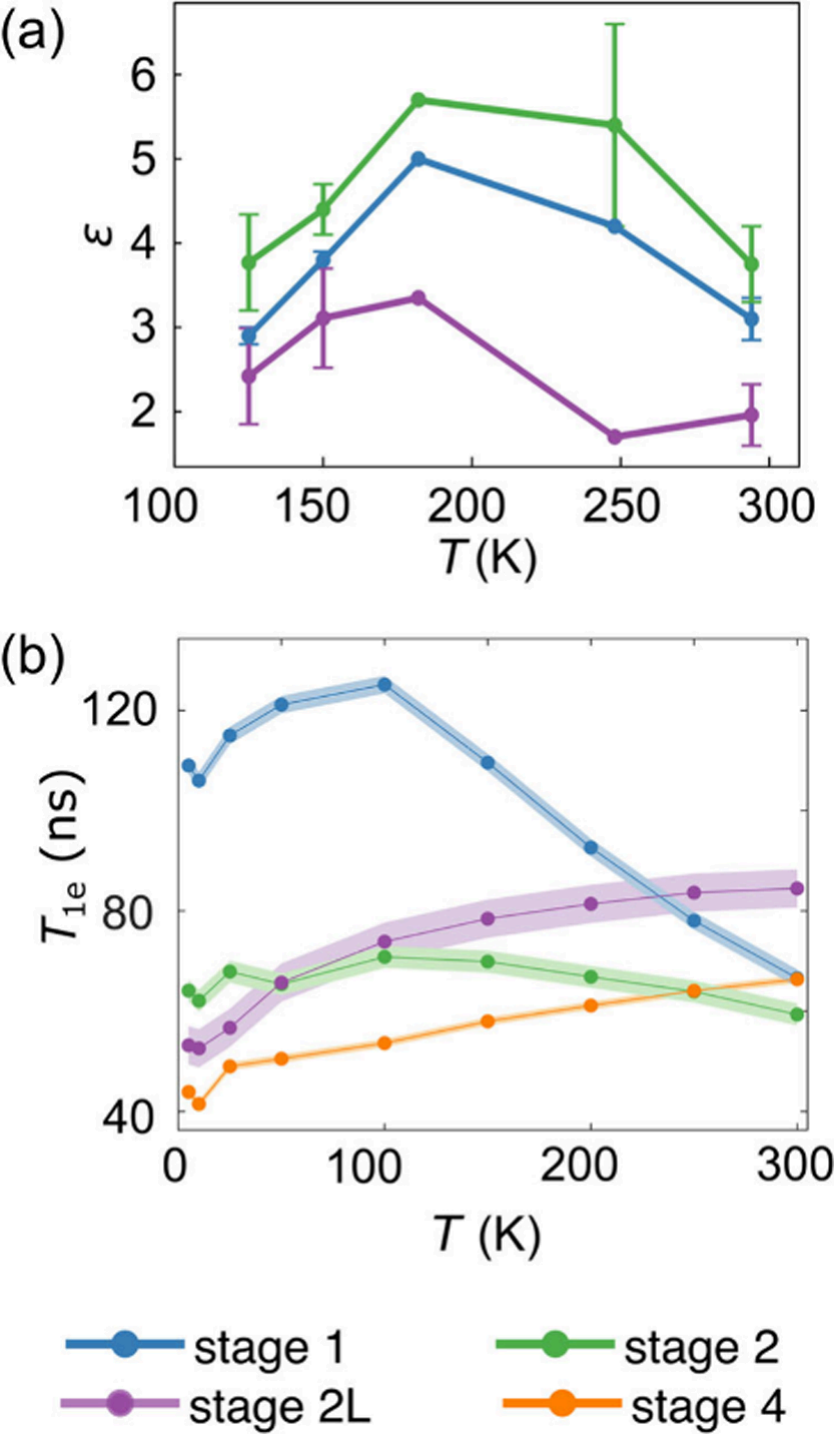
(a) Maximum ^7^Li enhancement of the intercalated Li peak (by intensity) for the stage 1, 2, and 2L graphite samples, measured via Hahn echos at different temperatures in the 100–300 K range (with sample temperatures measured with KBr), spinning at 20 kHz, and diluted with quartz powder (95 wt %). Error bars were determined by repeating the measurement with a second sample with same lithiation and dilution ratio. Some repeats were not possible due to experimental constraints and so no error bars are given. (b) The temperature dependences of the *T*_1e_s for the four graphite stages, reproduced from ref 24 (Copyright 2023, American Chemical Society) under Creative Commons license CC-BY 4.0.

To explore the role of the electron relaxation times on the observed DNP enhancements, variable temperature ^7^Li enhancement profiles were measured for three different samples containing the two dense stages and for dilute stage 2L (Figure 5(a) electrochemistry shown in SI Figure S2, spectra shown in Figure S3). For both dense stages, the enhancement reaches a maximum as the temperature decreases in the range 100–300 K, but the maximum does not coincide with that of the *T*_1e_ measurements (100 K); rather, it occurs at ∼170 K for stage 1 and ∼250 K for stage 2. The dilute stage 2L shows a more oscillatory behavior (likely due to the small enhancements measured, *ε* ∼ 1.8–3, increasing the error in the measurement, particularly with the unstable klystron^41^).

As explored further in the Discussion section, it is evident that the *T*_1e_ is not the only factor at play here and that additional quantities such as conductivity (and conductivity anisotropy), magnetic susceptibility and the nature of the hyperfine interaction (which changes with varying Li content, as shown in NMR by the different Knight shifts)^23,50^ are playing a role in the observed profiles.

The other parameter influencing the saturation factor is the microwave power. To probe saturation effects, the microwave power is typically ramped up, with the enhancement recorded for each power step. However, while gyrotron microwave sources offer a wide power range ∼5–22 W, the microwave power range in a klystron microwave source is between 1 and 5.2 W; in both cases, the effective power at the sample is smaller than the output power due to dielectric losses (sample and probe dependent and loss from coupling of source and probe) and skin effects in these metallic samples. The power limitations on the klystron mean that below 5.2 W little-to-no enhancement is obtained.

Rotor size also affects microwave penetration in the sample, with smaller rotors making microwave penetration more efficient. Thus, the effect of microwave power can be probed also (indirectly) through a change in rotor size. For instance, the enhancement for stage 1 Hitachi graphite in a 3.2 mm probe using a sapphire rotor and at 5.2 W only reached a maximum of ε ∼ 1.5 (Figure S4), while in the 1.3 mm probe the maximum observed was ε ∼ 5. Probe design is also important, the 1.3 mm probe used here containing a mirror at the back of the stator which refocuses the microwaves onto the sample.^51^

A power curve was measured for the stage 1 sample at two different temperatures, 170 and 250 K, as well as different dilution ratios, using the larger range of power available at the gyrotron microwave source at the CRMN facility in Lyon (Figure 6). The signal enhancement increases with microwave power but appears to “saturate” when not even the maximum available power was used. It is unlikely that the EPR transition was saturated at this power range—since no shift of the ^7^Li signal is seen—but instead it is possible that at higher power heating effects are more pronounced, resulting in shortening of the *T*_1e_ and therefore lower enhancements, as it was previously observed by Svirinovsky-Arbeli and co-workers, although using an exogenous DNP approach, for a range of conductive carbons.^52^ Therefore, it appears that while high microwave powers help in obtaining larger ^7^Li enhancements in lithiated graphite, too high a power likely results in sample heating and reduces the observed enhancements.

**Figure 6:**
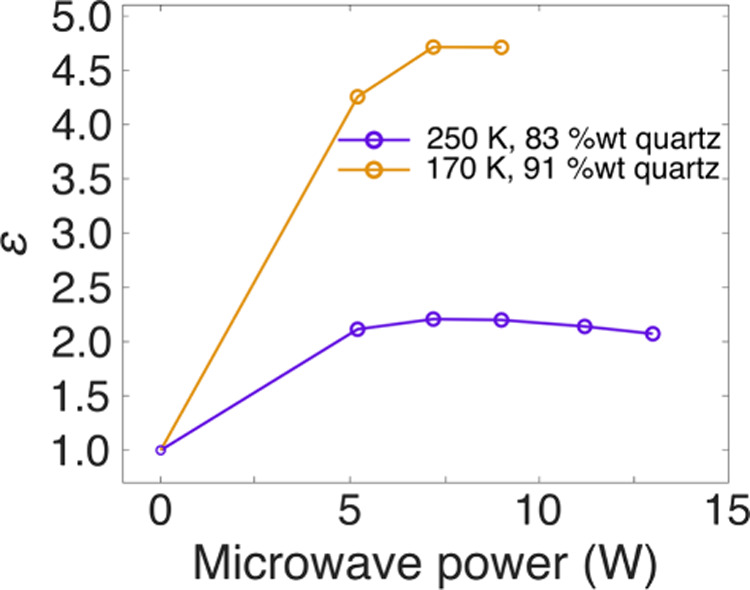
Enhancement by area of the intercalated Li signal (42.6 ppm) in LiC_6_ (packed in an 1.3 mm rotor) as a function of applied microwave power when using a gyrotron microwave source at 170 and 250 K. The enhancement of 1 at 0 W corresponds to the microwave OFF spectrum, with the enhancement being normalized with respect to that. The spectra were recorded with a one pulse sequence, with a recycle delay of 10 s. The reported temperatures are sample temperatures in the absence of microwaves (some heating is expected with microwaves on).

One last factor linked to microwave penetration is the effect of sample dilution, which was briefly touched upon above. This will be explored in more detail below in the context of skin effects. These skin effects also limit the total enhancement of the Li signal.

#### Coupling Factor

The coupling factor expresses the efficiency of the cross-relaxation events against other relaxation processes. Cross-relaxation events involve the simultaneous flipping of both coupled electron and nuclear spins, with ZQ corresponding to a flip-flop transition and DQ corresponding to a flop–flop transition (Figure 1). A scalar, through bond (Fermi contact-like) interaction can only induce ZQ cross-relaxation, while a dipolar hyperfine interaction can induce both. From the expression

5 $$\xi =\frac{W_{2}-W_{0}}{W_{0}+2W_{1e}+W_{2}}$$

it can be seen that a larger difference between DQ and ZQ cross-relaxation rates (*W*_2_ and *W*_0_, respectively) results in higher observed enhancements.^31,53–55^ If the scalar hyperfine interaction dominates, as in Li metal, *W*_1e_ *= W*_2_ = 0, and ξ = −1. If the dipolar interaction dominates, ξ = 0.5, *i.e.* negative enhancements are observed with half the theoretical magnitude that could be obtained for a scalar-only hyperfine interaction. The enhancement observed in Li-intercalated graphite is positive, suggesting that the scalar component of the hyperfine interaction dominates. However, the smaller magnitude enhancement as well as the presence of an anisotropic component to the hyperfine coupling tensor (*i.e.*, a dipolar hyperfine interaction) observed in the HFEPR spectra^24^ suggest that dipolar contributions may be present that decrease the overall polarization efficiency.

In Li-intercalated graphite, the degree of metallicity can be tuned depending on the lithiation stage, with the dense stages being more conducting than the dilute stages, as seen in EPR (through tracking the asymmetry in the CW absorptive line shape,^24^ see below) and in NMR (by tracking the magnitude of the ^7^Li Knight shift).^23,50^ The effect of metallicity on DNP enhancement can be inferred from Figure 5(a). Perhaps unsurprisingly, stage 2L produced low enhancements, likely due to the lower conductivity, producing a poor match with the cross-relaxation rate. In contrast, stage 2 is more efficient than stage 1 across all temperatures. This could be explained by considering the temperature dependence of the metallicity (tracked previously by EPR),^24^ with stage 2 appearing more conducting at *T* < 180 K, possibly indicating a better match of the time scales in that stage. The Knight shift for stage 2 is also slightly larger, indicating a greater density of states at the Fermi level at the Li 2s orbital, the resulting hyperfine interaction providing a stronger driving force for relaxation.

Another factor that should be considered is the strong anisotropy of the EPR signal. The maximum in the EPR signal originates from parts of the powder that are oriented with the normal to the carbon’s basal plane perpendicular to the static magnetic field (*i.e.*, with the planes aligned parallel to the field). Thus, for a static DNP experiment, we would not expect the whole line shape to be uniformly excited. Our DNP experiments are performed under MAS, and the orientations of the graphitic sheets become time dependent. Thus, although the applied microwave frequency may not match the EPR transition for a specific orientation of the powder, a larger fraction of the sample will sweep through the conditions under which the EPR transition frequency matches the microwave frequency. A DNP enhancement will result, but nonetheless, this effect may reduce the overall enhancement. The enhancement dependence on the spinning speed is shown in Figure S5.

#### Skin Effects and EPR Spectroscopy

Microwave penetration in metals is limited to within their skin depth (a phenomenon known as the skin effect).^48,56,57^ Skin effects therefore limit the number of electrons that can be excited by microwave irradiation since only those electrons within the skin depth, δ, “see” the microwaves at any given point. Furthermore, the electrons can travel outside the skin depth on the time scale of the NMR experiment—their magnetization may be lost to the bulk and may not be observable.

Microwave penetration can be improved by changes in the hardware (such as the aforementioned improved performance of the 1.3 mm probe vs 3.2 mm probe) as well as changes to the measurement conditions and the sample itself. For example, lower temperatures, while increasing the *T*_1e_, also increase the electrical conductivity of the sample (via reduction of scattering via phonon modes, etc.), therefore decreasing the skin depth. On the other hand, at higher temperatures, heating effects caused by interaction of the microwaves with carbonaceous materials become more pronounced; furthermore LiC_6_ degrades over time at *T* > 60 °C, due to reactions with the SEI, as seen recently in our NMR experiments.^23^ Therefore, intermediate temperatures are preferred.

DNP samples can also be “diluted” with a dielectric material (commonly KBr, here quartz powder) which is ground together with the sample and reportedly results in higher DNP enhancement of e.g. biradical solutions.^58^ Since particles like KBr are less lossy—and, particularly relevant to this system, not metallic—the microwaves penetrate the samples more easily, with the distributions in KBr particle sizes resulting in scattering of the microwaves, which are therefore more uniformly distributed within the sample.^58^

A similar approach was taken here, where quartz powder was used as diluent instead of KBr due to the observed sample degradation on contact of LiC_6_ with KBr. The dilution ratio was varied between 0 and 90 wt % quartz and the resulting powders were packed in 1.3 mm rotors and first measured by EPR spectroscopy, since we have previously shown that the EPR spectra of these samples provide a relatively straightforward method to measure the extent of microwave penetration.^24^ It is clear that dilution affects the asymmetry of the cw EPR absorption lineshapes (Figure 7(a)). The distorted lineshapes are known as Dysonian lineshapes, with higher degrees of metallicity and smaller skin depths giving rise to larger *A*/*B* ratios, (*i.e.*, the ratio of amplitude of the left positive peak *A* to the right negative peak *B* in the derivative of the absorption resonance). From inspection of the lineshapes, pure (nondiluted) LiC_6_ powder packed in a rotor produces a more Dysonian line shape than when diluted with quartz (which has a similar effect to the loose packing of LiC_6_ powder in a capillary, cf. ref. 24).

**Figure 7:**
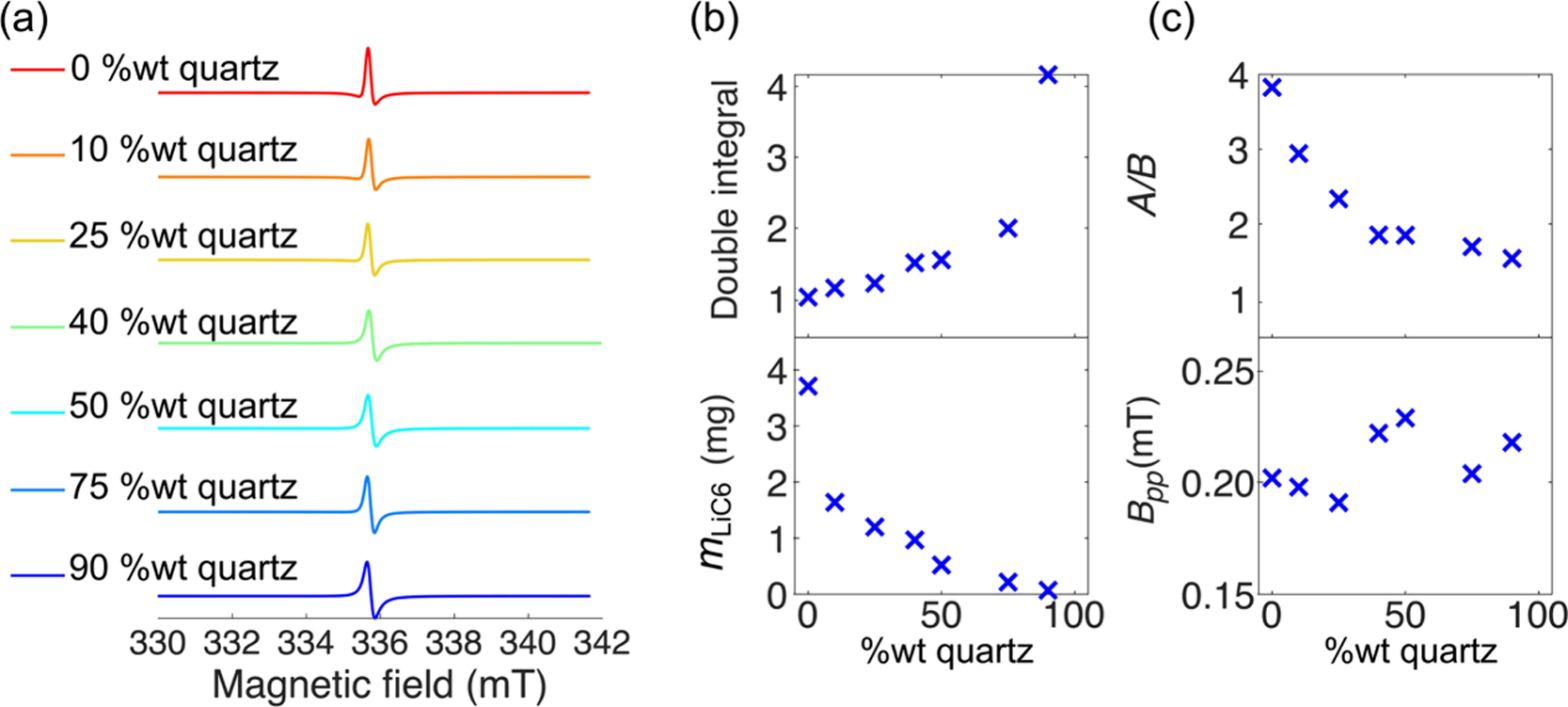
(a) X-band EPR spectra of LiC_6_ diluted with a quartz powder and packed in a 1.3 mm rotor at room temperature. The asymmetry in the line shape decreases as the wt % of quartz in the mixture increases. (b) Double integral of the CW spectra scaled by the mass of graphite (top) vs dilution (wt % of quartz); a more intense normalized graphite signal is seen for higher dilution factors. The bottom shows the approximate mass of LiC_6_ in the rotors measured. (c) Variation of the *A*/*B* (asymmetry) parameter vs dilution (wt % of quartz) (top); peak-to-peak line width (bottom) vs dilution (wt % of quartz) indicating that dilution does not affect the intrinsic electronic properties of the material but only microwave penetration in the sample.

The decrease in the *A*/*B* parameter (and increase in signal intensity when normalized for the total carbon content, Figure 7(b)) on dilution is ascribed to a reduction of the sample’s *effective* thickness which varies as the ratio of the particle diameter *d* and skin depth δ, *i.e.* *d*/*δ*, resulting in less pronounced skin effects and therefore in more electrons being detected. It should be noted that the sample’s skin depth does not change on dilution as it is a property intrinsic of the material (it depends on the conductivity); it is rather the effective particle size which varies—from the individual particles in the highly diluted case to the larger particle agglomerates seen in the less diluted scenarios. The effect of dilution on the EPR spectra was therefore to increase the overall number of spins that are excited and detected and ultimately to reduce skin effects. A confirmation that dilution does not change the properties of the LiC_6_ powder is in the negligible variation in the EPR line width, which oscillates around 0.2 mT (Figure 7(c)).

In our previous work^24^ we estimated a skin depth perpendicular and parallel to the *ab* planes of LiC_6_ of 4 and 1 μm at the X-band magnetic field strength, the differences in these values arising from the large anisotropy in the conductivity of this material.^24,25,27^ Using a skin depth of 4 μm and an average particle diameter of 13 μm produces a *d/δ* ratio of approximately 3.25 and (from Figure 15 in ref 59) an estimate for the *A*/*B* ratio of approximately 2, following a similar analysis to that used in refs. 24,59 Using instead the smaller skin depth (1 μm) yields an even larger *d*/*δ* ratio (13) and a larger estimate for the *A*/*B* ratio (the exact value depending on the relative time scales of *T*_2e_ relaxation and diffusion in and out of the skin depth, *T*_D_).^59^ The graphite particles are not, however, spherical, and for the Hitachi sample, they show considerable preferred orientations, with the *ab* planes of the more ordered graphite particles aligning with the substrate. The thickness of these particles is likely not accurately measured via our analysis of SEM data, and this very qualitative analysis of the *A*/*B* ratios suggests that they are thinner than 13 μm. Furthermore, lower *A*/*B* ratios (∼1.65) are obtained in the 90% diluted sample (Figure 7(c)), corresponding to a *d*/*δ* ratio of ∼2.5 and a particle size ranging 2.5–10 μm, depending on the chosen skin depth—*i.e.*, the majority of the particles can be uniformly irradiated if the LiC_6_ powder is sufficiently diluted.

The ramifications of this phenomenon were then explored in DNP (Figure 8) using a LiC_6_ sample diluted with quartz in a 1.3 mm rotor, at 250 and 100 K and using a klystron microwave source. It can be readily seen that substantial levels of dilution (>85%) are required to observe any DNP enhancement at all, with a plateau being reached when >90% of the sample mass is quartz (at 250 K), suggesting that dilution helps microwave penetration to an extent until it stops having any further effect. This is also easily explained by considering the role of the quartz particles in the sample: they break up the particle agglomerates, but the graphite intrinsic particle size distribution remains, which means that skin effects remain present for some of the larger particles. At lower temperatures, where the conductivity is higher and the skin depth is even smaller, further dilution does result in higher enhancements. Note that while skin effects can largely be overcome for the diluted samples at X-band (∼9.5 GHz), the estimated skin depths for graphite at the DNP field strength (∼263 GHz) drop to ∼0.2 μm and ∼0.7 μm parallel and perpendicular to the basal planes, respectively. Therefore, skin effects remain pronounced at higher static magnetic fields.

**Figure 8:**
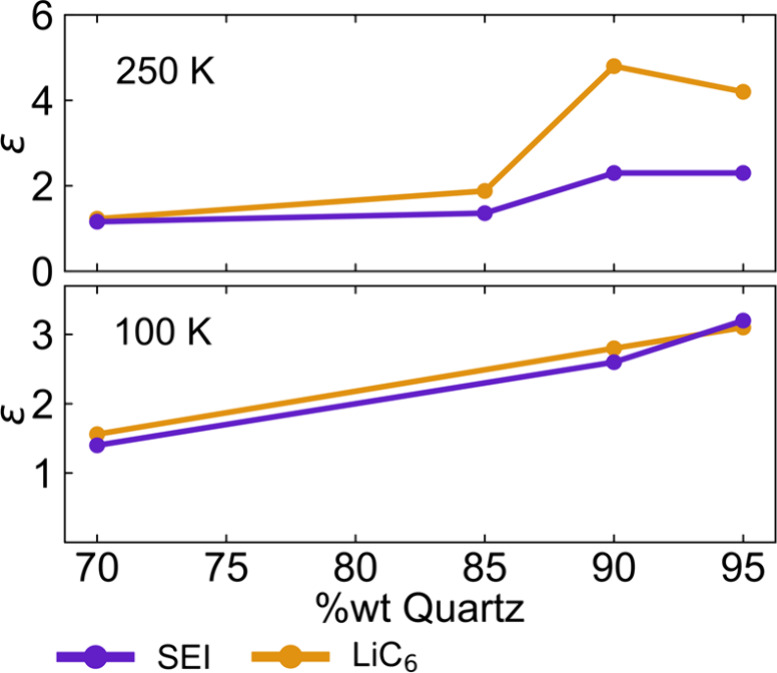
Effect of sample dilution on the observed enhancement of the Li SEI and LiC_6_ peaks. The enhancements were measured at 250 K (top) and 100 K (bottom) on LiC_6_ samples packed with quartz in 1.3 mm rotors, spinning at 20 kHz, and using a klystron microwave source (5.2 W).

### Effect of Different Graphite Morphologies

#### ^7^Li DNP Enhancement

^7^Li DNP spectra were taken of the five LiC_6_ samples whose electrochemistry and particle sizes are shown in Figure 3 and Table 1, respectively. All samples, except the BM graphite, were measured at 170 K and using a gyrotron microwave source. The BM sample could only be measured at room temperature (RT) and using a klystron microwave source due to experimental constraints; the sample degrades too quickly to be shipped. The ^7^Li ON/OFF spectra are shown in Figure 9, with the enhancements reported in Table 1. For all spectra, two main features are seen: a peak at 42.6 ppm corresponding to Li ions intercalated in the graphite (the LiC_6_ peak) and a peak at ∼ 0 ppm corresponding to the diamagnetic degradation products in the SEI. For all graphites except the BM sample, the LiC_6_ peak is more intense as usually there are more Li ions in the bulk Li than in the SEI; the BM sample, however, has a higher surface area compared to the other graphites (based on the SEM analysis, Figure 2(e)), resulting in more electrolyte degradation occurring on the first lithiation as seen via the extremely high capacity of close to 250 mAhg^–1^ between 1 and 0.5 V on lithiating this sample electrochemically (Figure 3).

**Figure 9:**
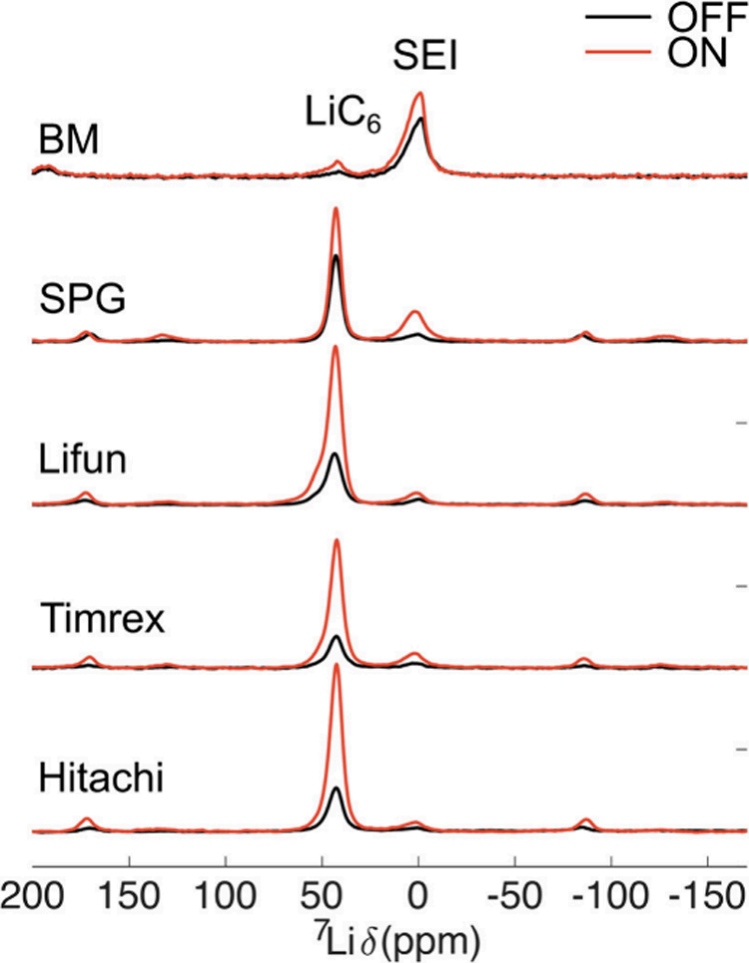
^7^Li DNP ON/OFF one pulse spectra of the 5 different graphites examined here. The ON/OFF spectra are normalized by the number of scans acquired. The recycle delay was 10 s for the experiments performed at 170 K (Hitachi, Timrex, Lifun, and SPG) and 5 s for those at 290 K (BM), quantitative for the intercalated Li peak. The samples at 170 K were spun at 20 kHz, while the sample at 290 K was spun at 30 kHz. All samples were diluted with 90 wt % quartz. The enhancement factors for the intercalated Li peak and the SEI peak are reported in Table 1.

In all graphite samples except SPG, the intercalated Li peak is enhanced more than the SEI peak—consistent with a model where hyperpolarization is spin-diffusion limited, with the polarization having to “travel” from the bulk into the surface phases. The Hitachi and Timrex showed the highest enhancements (*ε*_LiC6_ = 4 and *ε*_SEI_ = 3), followed by the Lifun sample (*ε*_LiC6_ = 3 and *ε*_SEI_ = 2). Perhaps surprisingly, the SPG and BM graphites, both with smaller particle sizes and size distributions, and therefore less significant skin effects, had lower enhancements (*ε*_LiC6_ < 2.3). Furthermore, in the SPG graphite, the SEI signal was enhanced more than the bulk LiC_6_ signal, which is surprising and might be therefore indicative of more localized hyperpolarized spins at a more defective or disordered surface. While DNP mechanisms involving chemical exchange between the metallic Li component and SEI have been proposed for Li dendritic samples,^60^ our measurements were performed at 170 K where this mobility will be reduced.

A preliminary examination of DNP enhancements of the different graphite samples at different extents of lithiation reveals the somewhat unexpected enhancement of an ∼8–15 ppm of ^7^Li signal in some nominally stage 2 samples, Figure 10. This signal is not due to the stage 2 regions, but is instead attributed to some residual dilute stage phase, which could have formed due to sample degradation or due to incomplete lithiation of parts of the sample. It is, however, only visible under microwave irradiation and has a DNP enhancement of greater than 6 (the lower limit being estimated from the noise level in OFF spectrum), the maximum occurring at a slightly lower magnetic field than the enhancement of the dense stages, consistent with the larger *g*-factor of the dilute stages compared to dense stages (Figure S6).^24^ The larger enhancement observed here compared to the dense stages suggests that the enhancements of these intermediate stages (between 2L and 2) could possibly be ascribed to different cross-relaxation kinetics (due to different electron mobilties) or the combination of high conductivity in one phase (dense stage 2) providing the Overhauser mechanism and the lower conductivity in the second (2L), providing a greater skin depth (and thus greater microwave penetration).

**Figure 10:**
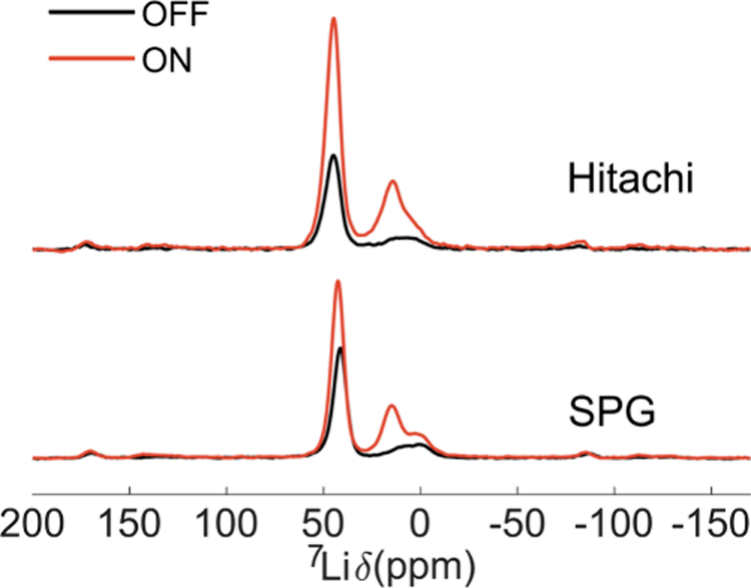
Examples of slightly degraded stage 2 samples of two different graphites, Hitachi MagE3 and SPG, which show a high DNP enhancement of a peak at 8–15 ppm, attributed to residual dilute stages. These spectra were measured as Hahn echo, with a recycle delay of 10 s at 250 K, under MAS rate of 20 kHz, using a dilution factor of 95 wt % with quartz.

#### X-band and High Frequency EPR

Since the hypothesis that a smaller particle size could improve DNP enhancements through irradiation of the entire particle (*i.e.*, the scenario where *d* = or < δ) did not prove correct, the different LiC_6_ graphites were then investigated by EPR to understand their local electronic structure better.

The X-band EPR spectra, recorded at RT in capillaries, for the 5 different graphites are displayed in Figure 11(a). The Hitachi, Timrex, and Lifun show a very similar EPR signal at X-band, with line width ∼ 0.2 mT (increasing slightly across the series, Figure 11(b), Table 1), while SPG and BM have noticeably broader line shape (>0.5 mT). In particular, the very broad line shape of the BM sample is consistent the considerable disorder that has resulted from ball-milling.

**Figure 11:**
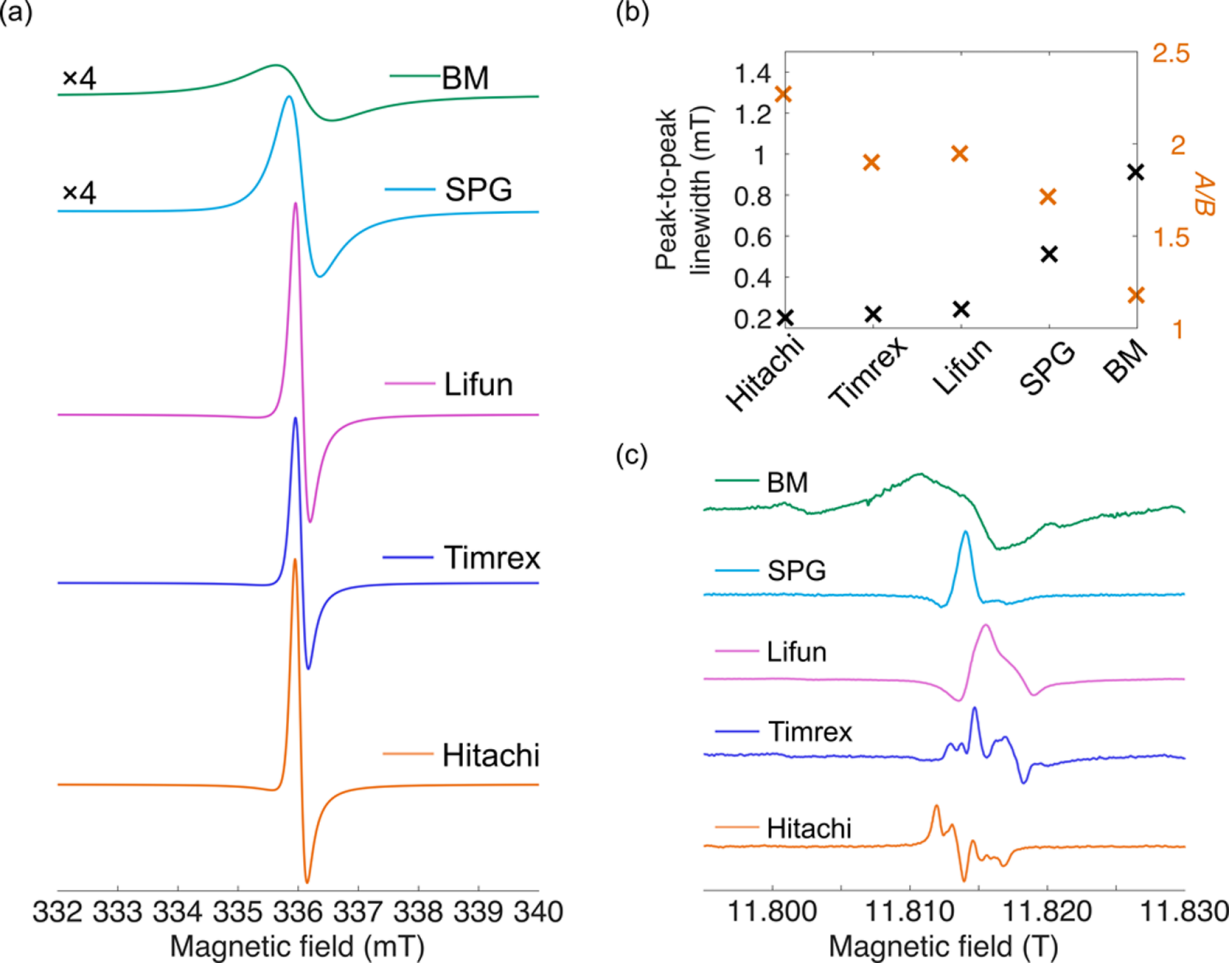
(a) Continuous wave EPR spectra of the LiC_6_ Hitachi, Timrex, Lifun, SPG, and BM graphites recorded at the X-band and at RT with a 1 mW microwave power and 0.1 mT modulation amplitude. The spectra are scaled by sample mass in the capillary, and the samples were not diluted with quartz. (b) Variation of the peak-to-peak line width and asymmetry parameter (*A*/*B*) across the five graphites. (c) High frequency EPR spectra of the five graphite samples measured at 331 GHz and at 50 K.

The *A*/*B* asymmetry parameters (from powders measured in capillaries) are similar, and around *A*/*B* ∼ 2 for the first three graphites, Hitachi, Timrex, and Lifun, with lower values (*A*/*B* ∼ 1.7 and ∼ 1.2) for the remaining two graphites, indicating the former set have similar *d*/δ ratios, while those of the SPG and BM samples are smaller. While the analysis of the effect of dilution on the *A*/*B* ratios performed for the Hitachi graphite sample shows that dilution is critical to determine the “true” skin depth, as opposed to one dictated by the agglomerates that are inevitably present in the nondiluted samples, the decrease in *A*/*B* ratios largely tracks the average particle sizes (noting the broad distributions in sizes; see Table 1). In the SPG and BM graphites, the particle size is small (<9 μm) and the particle size distribution is narrower (Table 1), consistent with the smaller *A*/*B* ratio and the ability of the microwaves to irradiate a larger fraction of each particle (*i.e.* *d*/δ is small). The differences in morphology and in any treatment/processing the graphites underwent (especially SPG, which was spherodized and coated) will also likely affect conductivity. In addition, the presence of defects strongly affects microwave absorption as seen for (semi)­conducting carbons, where significant heating effects have been observed.^52^

The local structure of these graphites was also investigated at high frequency (331.2 GHz) and at 50 K (Figure 11 (c)); the figure includes a reproduction of the HFEPR spectrum of the Hitachi graphite analyzed in ref. 24.

Following the approach used in our previous work^24^ to fit the Hitachi sample, three axial components (which we assigned to coupling to 2Li, 1Li and 0Li) were fit to each signal (Figure 12, Table 2), with similar magnitude hyperfine couplings (given as ranges to reflect the error in the fit). (Note that in our previous work, we showed that the sizes of the hyperfine coupling constants extracted from these fits were consistent with the values of the Knight shifts seen for the ^7^Li nuclei in LiC_6_/LiC_12_, the Knight shifts also being a measure of the electron–nuclear hyperfine interactions in a metallic system, justifying our assignmnents). The main difference is in the ratio of the three components, with the 0Li component being present at much higher percentages than in the Hitachi graphites. For the SPG graphite, this can be rationalized by considering the spherodization treatment that natural graphites receive, which results in the particles being covered in amorphous C and any pores being filled. The average particle sizes are also smaller meaning there are likely more surface spins that are not within the ordered LiC_6_ crystalline regions. For the Timrex graphite, the increase in defect spin component may be due to its particle morphology, which consists of several small particles agglomerating around a bigger particle—something occurring also to an extent in the Hitachi sample, but the smaller particles here are typically >3 μm, in contrast to <1 μm for the Timrex sample (Figure 2(a-b)). The role of electron mobility and self-decoupling also needs to be considered in these samples, as electron mobility is likely faster than the time scale of the hyperfine interaction, however, this phenomenon is likely to be strongly affected by particle size and defect concentration.^59^

**Figure 12:**
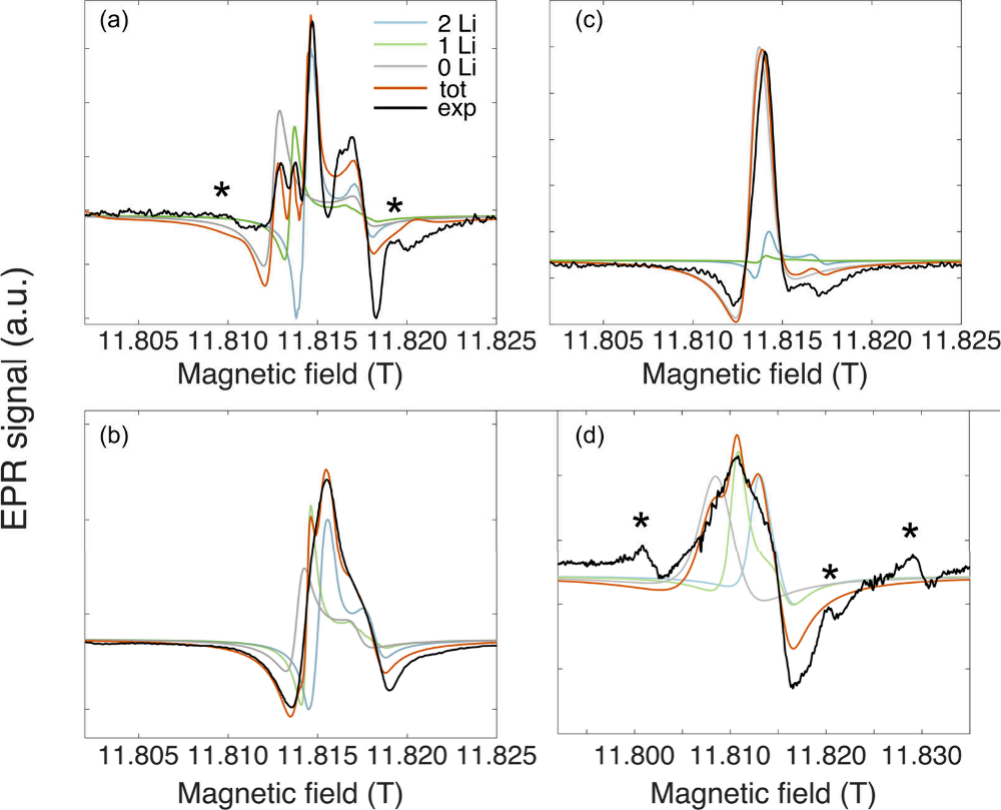
High frequency EPR spectra and the respective fits recorded at 331.2 GHz and at 50 K of LiC_6_. (a) Timrex graphite, (b) Lifun graphite, (c) SPG graphite, and (d) BM graphite. (a) and (d) also show the presence of Mn^2+^ impurities in the quartz capillaries (denoted by *). In (d) one of the Mn^2+^ hyperfine peaks falls in the middle of the graphite signal—its contribution to the total intensity was accounted for in the fit.

**Table 2:** Axial *g*-Tensors Component as Deduced from Fitting the HFEPR Spectra of the Hitachi, Timrex, Lifun, SPG, and BM Graphites; Weight Percentages of the Different Components; Isotropic *g*-Values Obtained at the X-band at Room Temperature (RT); and Isotropic Hyperfine Coupling Constants for the Two Different Components, 2Li and 1Li, Given as Ranges to Reflect the Error in the Fits^a^

	2Li component			1Li component			0Li component				
Graphite type	*g*_*x*__*,*__*y*_	*g*_*z*_	%	*g*_*x,y*_	*g*_*z*_	%	*g*_*x,y*_	*g*_*z*_	%	*g* (X band, RT)	*A*_*iso*_, MHz
Hitachi	2.0043	2.0036	56	2.0044	2.0037	31	2.0045	2.0042	13	2.0133	4.6 (2Li)
											3–5 (1 Li)
Timrex	2.0041	2.0035	29	2.0042	2.0036	19	2.0044	2.0036	52	2.0132	4.5–5.5 (2Li)
											3–5 (1 Li)
Lifun	2.0039	2.0035	36	2.0041	2.0035	27	2.0042	2.0036	37	2.0131	4.5–5.9 (2Li)
											3.5–7.0 (1Li)
SPG	2.0041	2.0036	6	2.0042	2.0037	16	2.0043	2.0041	67	2.0129	4–5 (2Li)
											2.6–6.6 (1Li)
BM	2.0043	2.0039	19	2.0047	2.0039	27	2.0051	2.0047	54	2.0131	2–7 (2Li)
											1–6 (1Li)

a No hyperfine interaction to Li is seen for the 0Li environment. The data for the Hitachi is the same shown for stage 1 in ref. 24 and is reproduced here for convenience.

Comparing the ratio of the 2Li:1Li components across the Hitachi:Timrex:Lifun series, 1.8:1.5:1.3, shows that the 1Li component increases with a decreasing particle size. Smaller particles are more easily lithiated,^61^ reducing the concentration gradient observed in the Hitachi graphite and generating a more homogenized distribution of Li ions within the structure. Corroborating this, the *g*-anisotropy and *g*-factors of the three components are more similar (Table 2). It is also important to stress that at the higher fields used to extract the 2Li:1Li:0Li components, the skin depth will be much smaller than that for the X-band spectra (but closer to that of the DNP experiments)—at 331 GHz (HFEPR frquency used here), δ = 0.17 μm parallel to the basal planes and δ = 0.65 μm perpendicular to the basal planes. Hence, the 2Li:1Li:0Li ratios given in Table 2 will be weighted toward species that are found closer to the surface and within the skin depth.

### OEDNP of a Wider Range of Nuclei in Lithiated Graphite

The Overhauser effect in lithiated graphite anodes could in principle involve the three nuclei present in the bulk, ^6,7^Li, ^13^C, and any ^6,7^Li-containing surface SEI species, which can be potentially be reached by spin diffusion, chemical exchange, and via a direct polarization mechanism. The polarization thus achieved can then be transferred to, for example, ^1^H-containing compounds at the surface via cross-polarization experiments. The OE efficiency in enhancing ^6,7^Li, ^13^C, and ^1^H signals is discussed below, with samples prepared from Hitachi graphite.

The signal with the highest enhancement was the bulk intercalated Li signal in both ^6,7^Li spectra, with larger enhancements being observed for ^6^Li, due to its lower gyromagnetic ratio (eq 1, Figure 13(a)). A lower estimate for the enhancement of *ε* > 8 at RT was estimated from the noise level of the OFF spectrum, since a signal cannot be seen above the noise for the spectrum acquired using natural abundance Li (Figure 13(a), cf. *ε* ∼ 3 for ^7^Li at RT). Again both bulk (intercalated Li) and surface Li species (SEI) are enhanced. The mechanism resulting in the SEI signal enhancement is not fully understood, with four different hypotheses currently proposed in the literature (for the Li metal case): polarization via spin diffusion from the bulk; via chemical exchange with the bulk; via direct polarization through the dipolar hyperfine interaction and through the Fermi contact interaction. Since the observed enhancement is positive in sign, direct polarization through the dipolar mechanism is excluded, as the enhancement would have been negative in that case. Spin diffusion is driven by homonuclear coupling, so its efficiency is expected to decrease with nuclei with lower natural abundance and gyromagnetic ratios—*i.e.* ^6^Li has a less efficient spin diffusion path than ^7^Li and therefore requires longer delays for the polarization to be relayed.^62^

**Figure 13:**
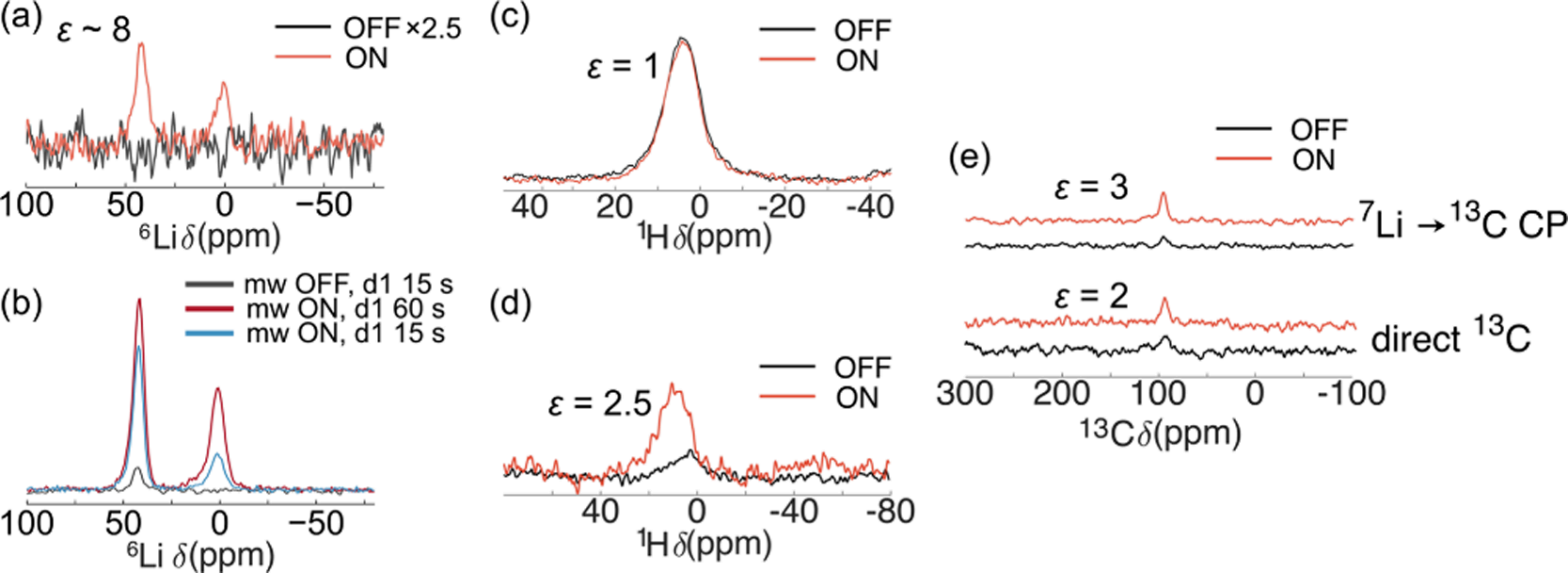
(a) ^6^Li DNP ON/OFF Hahn echo spectra of natural ^6^Li abundance LiC_6_ diluted with quartz (95 wt %) and spun at 25 kHz at 300 K (sample temperature, determined *ex situ* from the ^79^Br shift of KBr). The recycle delay was 60 s for both spectra. The mw OFF spectrum was measured over 10.5 h while the mw ON spectrum was recorded over 4.25 h. (b) ^6^Li DNP ON/OFF Hahn echo spectra of ^6^Li-enriched LiC_6_ diluted with quartz (95 wt %) and spun at 25 kHz at 300 K. To achieve isotopic enrichment the sample was cycled against ^6^Li metal. d1 in the legend indicates the recycle delay used. (c) ^1^H DNP ON/OFF spectra of LiC_12_ showing no enhancement at 170 K using a klystron microwave source, spinning at 20 kHz, with a recycle delay of 5 s; (d) ^7^Li → ^1^H cross-polarization (CP) DNP spectra measured at room temperature, using a recycle delay of 5 s and a contact time of 700 μs. (e) ^7^Li → ^13^C and direct ^13^C DNP spectra for a LiC_6_ sample measured at 170 K, spinning at 20 kHz, with a recycle delay of 10 s and a contact time of 8 ms for the CP, using a gyrotron microwave source. All experiments were performed with samples prepared from Hitachi graphite.

The ^6^Li SEI signal could only be detected in the sample prepared with natural abundance Li in a ^6^Li DNP experiment when a recycle delay of 60 s was used (see Figure S7 and Figure 13(a)) and not with a 15 s recycle delay. The SEI signal could, however, be observed when these experiments were repeated with a ^6^Li-enriched sample (therefore containing a more strongly coupled ^6^Li spin system, Figure 13(b)) with a recycle delay of 15 s, but the enhancement increases by a factor of ∼2 if the delay is increased to 60 s, in part due to the long recycle delays of ^6^Li spins and also the time taken for polarization to diffuse from the interface through the SEI. Exchange between the ^7^Li signals from bulk LiC_6_ and surface Li ions was not observed in EXSY experiments at room temperature over a range of mixing times (50, 100, and 1000 ms, see Figure S8), indicating that neither chemical exchange nor spin diffusion across the interface are strong drivers for generating polarization. We note that, in contrast to Li metal, chemical exchange between bulk and SEI Li cannot occur at all surfaces, e.g., the basal planes. Hence, and in contrast to Li metal, where rapid exchange has been seen,^60,63,64^ chemical exchange will likely be slower. The positive enhancement of the SEI signal is ascribed, at least in part, to direct enhancement via a Fermi contact interaction.

^1^H DNP spectra showed no enhancement (Figure 13(c)), even following a wide sweep of the microwave frequency (Figure S9), suggesting that there are no ^1^H spins at the LiC_6_/SEI interface. ^1^H enhancement could, in principle, occur via direct polarization, likely exploiting the dipolar hyperfine interaction, thus resulting in a negative enhancement: this was not observed. Alternatively, if unpaired electron density is present at the LiC_6_/SEI interface, weak hyperfine coupling to any protons located at this interface would produce positive enhancement and could then be propagated by (homonuclear) spin diffusion; however, this is also not observed here, again suggesting that no protons are present at the interface. Hyperpolarisation was transferred successfully instead to the surface ^1^H species *via* cross-polarization from the DNP-enhanced ^7^Li to the ^1^H nuclei (Figure 13(d)), in a similar way to that observed previously for Li metal.^19^

Direct ^13^C excitation of LiC_6_ also resulted in no enhancement at the powers offered by a klystron microwave source (max 5.2 W at the source), but at the higher power of the gyrotron source a small positive enhancement of 2 was measured (Figure 13(e, bottom)).

Analogously to the ^1^H spectra, ^7^Li → ^13^C CP was also successful in transferring polarization, resulting in an enhancement factor of 3 (Figure 13(e, top)). The need for higher powers when enhancing ^13^C can be explained in terms of the orbitals involved: while Li ions have unpaired electron density at the nucleus (in the valence *s*-orbital), the C valence band is comprised of *p*-orbitals, with a node at the nucleus. A negative Knight shift (arising from core polarization) results in a resonance at 96 ppm in the ^13^C NMR spectrum of LiC_6_,^23^ the (small) size of the shift suggesting little contribution of this valence band (containing unpaired electrons) to the density of states at the nucleus. A dipolar contribution is also likely, resulting a negative contribution to the enhancement.^23^

## Discussion

Enhancement *via* the Overhauser DNP mechanism proved successful in lithiated graphite anodes resulting in a modest direct enhancement of ^7^Li and ^13^C signals, as well as indirect enhancement (via CP) of ^1^H containing species in the SEI and of the bulk ^13^C intercalated graphite signal. The enhancement is positive in all cases, and the presence of only one maximum over a wide field range, as well as the field profile tracing the high frequency EPR line shape (Figure 4(b)), confirms that it originates from the Overhauser mechanism via a scalar-dominated cross-relaxation process.

It was shown, through variable temperature DNP measurements (Figure 5), that the enhancement is affected by several factors. Three main contributors to the enhancement were identified: namely, the coupling factor, the saturation factor, and microwave penetration; the leakage factor in these systems was found to be less important (*i.e.*, close to one).

The coupling factor, quantifying the relative efficiency of the ZQ vs DQ cross-relaxation process, is determined by the nature of the hyperfine interaction and the presence of spectral density (fluctuations—here the electron mobility) to dissipate the energy released by the cross-relaxation process. We showed via fits to the HFEPR spectra (Figure 12) that the hyperfine interaction between unpaired electrons and Li ions in lithiated graphite has both scalar and dipolar components, and thus both DQ and ZQ cross-relaxation transitions should be allowed. The presence of the two mechanisms results in smaller differences in the populations of the |*ββ*⟩ and |*βα*⟩ states—*i.e.* those that give rise to nuclear magnetization (Figure 1 and equation S1)—in comparison to that obtained if only one of the relaxation processes operated, and therefore less hyperpolarization is generated.

The graphites studied in this work are all battery grade materials and thus highly disordered. Furthermore, both the nature of this disorder and the morphology and sizes of the particles differ between samples, in part because of how they were processed for use in batteries. We have therefore explored a series of different parameters extracted from the EPR spectra, to attempt to determine some additional factors that affect the degree of enhancement (Figure 14).

**Figure 14:**
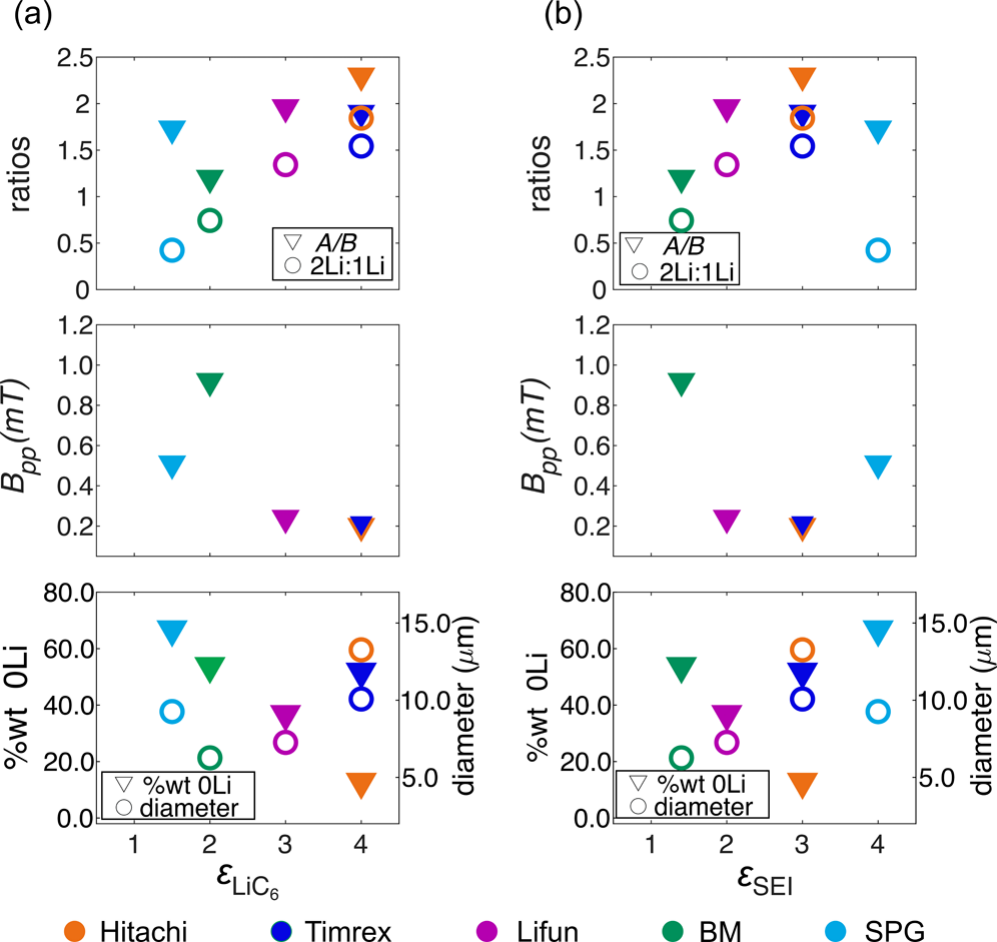
*A*/*B* ratio and ratio of the 2Li:1Li components in the HFEPR fits (top), peak-to-peak X-band EPR line width (*B*_pp,_ middle), and %wt of the defect component in the HFEPR fits and average particle diameter from SEM (bottom), all plotted as a function of the observed ^7^Li enhancement of the (a) LiC_6_ peak and (b) SEI peaks, as measured in the five different graphites examined here (Figure 9). For reference, ε_LiC6_ = 4 (Hitachi, Timrex), ε_LiC6_ = 3 (Lifun), ε_LiC6_ = 2.3 (BM), ε_LiC6_ = 1.5 (SPG), ε_SEI_ = 3 (Hitachi, Timrex), ε_SEI_ = 2 (Lifun), ε_SEI_ = 1.4 (BM), and ε_SEI_ = 4 (SPG).

The best fit of the components in the HFEPR spectrum were obtained when considering that the interaction with one nearby Li nucleus has a larger dipolar hyperfine coupling constant than the 2Li component. By taking the ratio of the relative contributions of the 2Li and 1Li components obtained from the fits, the effect of the dipolar hyperfine interaction on the enhancement can be at least qualitatively assessed (Figure 14, top, empty circles): high 2Li:1Li ratios are correlated with higher enhancements of the bulk signal, consistent with a weaker dipolar contribution to the hyperfine interaction and therefore a weaker contribution of the dipolar mechanism and thus a larger hyperpolarization buildup (enhancement). It is not, however, immediately obvious why the LiC_12_ sample results in a higher enhancement, since it should, assuming that our assignments are valid, contain more of the 1Li component.

For relaxation to occur, the hyperfine coupling should oscillate on a time scale of the order of the electron Larmor frequency, which, at 9.4 T, corresponds to a correlation time of ∼4 × 10^–12^ s. In metals, this time scale is roughly matched by the translational energy of the conduction electrons.^31,65^ In lithiated graphites, the metallicity can be tuned by varying the lithiation stage; *i.e.* different electron translational energies can be probed experimentally. Since the metallicity can be qualitatively assessed using the *A*/*B* asymmetry parameter from X-band EPR measurements, the *A*/*B* ratios were plotted against the observed enhancements (Figure 14, top, full triangles). There is a weak trend between a high *A*/*B* ratio and conductivity and enhancement, with SPG graphite being an outlier. We note, however, that the LiC_6_ samples were not diluted and they were only loosely packed in capillaries for the EPR measurements and it may be that the packing of this sample—with its very different morphology—was different, resulting in an anomalously high *A*/*B* ratio. Stage 2 (which is more conductive at low temperatures)^24^ consistently gives rise to better enhancements than stage 1 in the Hitachi graphite sample (Figure 5), and it may be that the effect of the conductivity outweighs the effect of the 2Li:1Li ratio discussed above.

The effect of the X-band EPR line width *B*_pp,_ and thus the *T*_2e_*, on the observed enhancements in these graphites is next explored in Figure 14, middle. On the assumption that *T*_1e_ = *T*_2e_ in metals, *B*_pp_ is a proxy for *T*_1e_ since $B_{pp}\propto \frac{1}{T_{2e}}$ : sharp line widths should be correlated with higher LiC_6_ enhancements. Again there is a weak trend consistent with this assumption with the BM sample being the exception. However, here the BM sample’s significantly broadened line width likely arises from a different source—namely the presence of a wide range of defects and localized spins introduced via ball-milling.

Lastly, the effect of particle size (and therefore microwave pentration and skin effects) is explored. Surprisingly, the larger graphite particles, in general, result in larger enhancements (Figure 14, bottom, open circles). While smaller particles increase the overall number of electrons that can be excited by the microwaves (as the skin depth and particle size become of similar magnitude), the increase in disorder and presence of grain boundaries, reducing the electronic conductivity, appears to result in less efficient coupling and saturation factors, resulting in lower enhancements.

The SEI enhancement is, in all but one case, smaller than the LiC_6_ bulk enhancement, with the exception being the SPG graphite. One source of the SEI enhancements involves a mechanism where polarization is built up in the LiC_6_ bulk and relayed to the surface SEI spins via spin diffusion or chemical exchange. However, EXSY experiments with variable mixing times (50 ms to 1 s, see SI Figure S8) did not show any cross-peaks, suggesting another mechanism is at play. The trends observed for the bulk LiC_6_ signal are largely reproduced in the SEI—namely that enhancement correlates with metallicity (*A*/*B* ratio) and 2Li:1Li ratio, with SPG graphite now being the sole outlier (Figure 14(b)). This sample has a large 0Li fraction (67%) arising from “defect” spins, as determined from the HFEPR spectrum of the SPG graphite (Figure 12(c), Table 2). At least some of these spins likely come from the spherodization and pitch coating applied to this graphite. It is therefore possible that these defect spins (located at the particle surface) are hyperpolarized and, being closer to the SEI, result in increased surface enhancement via a scalar mechanism. An initial investigation of the SEI showed no evidence of H atoms at the graphite/SEI interface as no enhancement of the ^1^H signal was observed. This suggests that species such as LiOH and LiH or even an organic carbonate are not directly present at the interface. Protonated species are found nearby in the SEI as the ^1^H signal can be enhanced via a CP experiment. The enhanced ^1^H signal is at a higher frequency than expected for hydroxides and hydrides,^66^ suggesting that they are organic in nature. A fuller analysis of these species will be the subject of future work.

Finally, it is still not clear how to reconcile the presence of resolved hyperfine interactions in the presence of mobile electrons, and it is possible that localized spins, or smaller domains, close to the surface of these graphites (and thus more effectively polarized/excited by the microwave irradiation in both high field EPR and DNP experiments) may play a role in electron localization. However, our assignment of 0Li to defective regions, rather than, for example, electrons delocalized in larger domains, is correlated with disorder, *not order*, in the carbons in support of our assignment. Further studies are required with a wider range of carbons and possibly with ^6^Li-enriched samples.

## Conclusion

The Overhauser DNP mechanism was introduced here as a way of polarizing the bulk and surface species in lithiated graphite. An overview of the mechanism was provided and contextualized in terms of lithiated graphite anodes and their differences in electronic properties, as compared to Li metal, where Overhauser DNP is more established.^19,21,22,60^ From the discussion above, it is clear that the principal limitations in the mechanism of the OEDNP in these materials are the short *T*_1e_s and the presence of both scalar and dipolar hyperfine couplings, reducing the overall coupling factor. Furthermore, the highly disordered graphite structure and the large microwave absorption of carbonaceous materials reduce conductivity and induce heating.

Substantial improvements in the observed enhancements were achieved by using high powers and smaller rotors as well as by diluting the conducting materials in quartz (improving microwave penetration). Variable temperature EPR studies revealed the need for intermediate temperatures (150–250 K) for optimum enhancements.

The analysis of different graphites showed that more ordered structures, with sharp EPR lines and higher conductivity, tended to have higher enhancements of the ^7^Li LiC_6_ signal. The SEI signal, by contrast, appears to be enhanced by defect, surface-based spins. Proton species in the SEI could not be observed in direct DNP but were observed in a ^7^Li → ^1^H CP experiment, providing a method to study the proton components in the SEI. Enhancement of ^13^C signals of the bulk were observed with sufficiently high microwave powers, slightly larger enhancements being obtained in the ^7^Li → ^13^C CP experiment, and potentially providing an approach to help assign ^13^C signals in more disordered carbons. Finally, the analysis performed above confirms the power of variable temperature EPR spectroscopy in determining the optimum conditions for DNP experiments, which was particularly useful when challenging and heterogeneous samples. Future extensions of the methodology presented here to study SEI formation are readily envisioned.

## Supplementary Material



## Notes

Overhauser DNP derivation, additional DNP NMR spectra (variable temperature, variable MAS rate), EXSY experiments, and electrochemical profiles of the different graphites (PDF)
