# Supplementary material for: Overhauser Dynamic Nuclear Polarization of Lithiated Graphite Anodes: Probing Bulk and Surface Structures
Source: Chem Mater. 2025 Jul 1;37(14):5167–82. doi: 10.1021/acs.chemmater.5c00845 (PMC12287998; doi:10.1021/acs.chemmater.5c00845)
Supplement: Supplementary file 1 [file cm5c00845_si_001.pdf]

Supplementary information

Overhauser Dynamic Nuclear Polarisation of lithiated graphite anodes: probing bulk and surface structures

Teresa Insinna,[1] Anne-Laure Barra,[2] Clare P. Grey[1],*

[1]Yusuf Hamied Department of Chemistry, University of Cambridge, Lensfield Road, Cambridge, CB2 1EW, United Kingdom

[2]LNCMI-CNRS, EMFL, Univ. Grenoble-Alpes, 25 Rue des Martyrs, B.P. 166, 38042 Grenoble Cedex 9, France

Contents

Table of Contents

Supplementary information ***1***

Contents 1

1 OEDNP derivation 2

2 Electrochemistry of the different graphite stages 5

3 DNP ON/OFF spectra of the different stages at various temperatures 6

4 ^{7}Li enhancement using a 3.2mm probe 7

5 Enhancement at different spinning speeds 8

6 DNP field profile for the lightly degraded stage 2 sample 10

7 Natural abundance ^{6}Li DNP spectra 11

8 ^{7}Li exsy spectra at variable mixing times 12

9 ^{1}H DNP field sweep 13

1 OEDNP derivation

An overview of the Overhauser effect is given now with some detail, although the reader is directed to refs[1–6] for a more in-depth discussion of the physics behind it.

Let us consider a coupled two-spin system comprising an electron *s* and a nuclear spin *I* with energy levels defined as m_s and m_I separated by the respective Larmor frequencies. Transitions between the electronic states rely on microwave irradiation at the (allowed) EPR transition, while transitions between nuclear states within the same electronic states rely on rf irradiation like in NMR. When continuous wave microwaves irradiate the EPR transition, the electron spins are excited to their excited states (W_{1e}). At this stage, different processes can occur (Figure S1): the spins can relax down to the ground states, producing no overall change (this process is therefore T_{1e} limited); the electron spins interact with the nuclear spins *via* the hyperfine interaction inducing spin flips of both electron and nuclear spins. The two possible relaxation processes are double quantum (DQ, W_2) cross relaxation, where $|\alpha\alpha\rangle \rightarrow |\beta\beta\rangle$, and zero quantum (ZQ, W_0) cross relaxation, $|\alpha\beta\rangle \rightarrow |\beta\alpha\rangle$. Whether DQ or ZQ relaxation occurs depends on the nature of the hyperfine interaction: a scalar, through bond (Fermi contact-like) interaction can only induce ZQ cross-relaxation, while dipolar hyperfine can induce both. If the rates of the DQ and ZQ relaxation processes differ, hyperpolarisation is built up and signal enhancement is observed.[7–9]

The changes in population of the different coupled electron-nuclear states can be described using the Solomon equations:[6]

$$\begin{aligned}
\frac{dN_{\alpha\alpha}}{dt} &= W_{1e} \cdot N_{\beta\alpha} + W_{1n} \cdot N_{\alpha\beta} + W_2 \cdot N_{\beta\beta} - (W_{1e} + W_2 + W_{1n}) \cdot N_{\alpha\alpha} \\
\frac{dN_{\alpha\beta}}{dt} &= W_{1e} \cdot N_{\beta\beta} + W_{1n} \cdot N_{\alpha\alpha} + W_0 \cdot N_{\beta\alpha} - (W_{1e} + W_0 + W_{1n}) \cdot N_{\alpha\beta} \\
\frac{dN_{\beta\alpha}}{dt} &= W_{1e} \cdot N_{\alpha\alpha} + W_{1n} \cdot N_{\beta\beta} + W_0 \cdot N_{\alpha\beta} - (W_{1e} + W_0 + W_{1n}) \cdot N_{\beta\alpha} \\
\frac{dN_{\beta\beta}}{dt} &= W_{1e} \cdot N_{\alpha\beta} + W_{1n} \cdot N_{\beta\alpha} + W_2 \cdot N_{\alpha\alpha} - (W_{1e} + W_2 + W_{1n}) \cdot N_{\beta\beta}
\end{aligned} \qquad \text{S1}$$

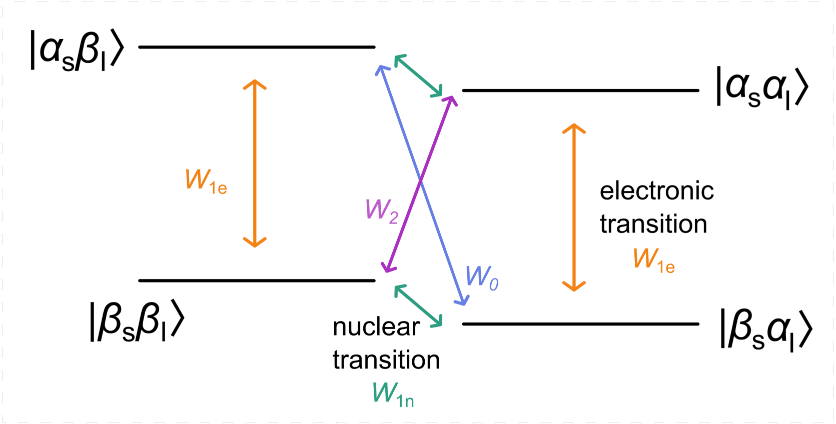

Figure S1. Reproduction of Figure 1 in the main text for convenience. Energy level diagram of a one electron (denoted by *S*)-one nucleus (denoted by *I*) system and the transitions that can occur in Overhauser DNP, with *α, β* denoting spin up and spin down configurations. W_{1e} is the rate of the EPR transition, ω_{1e}, W_{1n} is the rate of the NMR transition, ω_{1n}, and W_2 and W_0 describe the rates of the double quantum and zero quantum cross-relaxation transitions.

With $N_{\alpha\alpha}$, $N_{\alpha\beta}$, $N_{\beta\alpha}$ and $N_{\beta\beta}$ indicating the populations of spin states denoted by quantum numbers α, β in Figure S1. The nuclear and electron magnetisations in the *z*-direction are defined as

$$\langle I_z \rangle \propto \left(N_{\alpha\alpha} + N_{\alpha\beta}\right) - \left(N_{\beta\alpha} + N_{\beta\beta}\right)$$
$$\langle S_z \rangle \propto \left(N_{\alpha\alpha} + N_{\beta\alpha}\right) - \left(N_{\alpha\beta} + N_{\beta\beta}\right). \qquad \text{S2}$$

Combining Equations SS1 and SS2 and expressing the magnetisation upon polarisation in comparison to the equilibrium magnetisations I_0 and S_0 gives

$$\frac{d\langle I_z \rangle}{dt} = -(W_0 + 2W_{1e} + W_2) \cdot (\langle I_z \rangle - I_0) - (W_2 - W_0) \cdot (\langle S_z \rangle - S_0)$$
$$\frac{d\langle S_z \rangle}{dt} = -(W_2 - W_0) \cdot (\langle I_z \rangle - I_0) - (W_0 + 2W_{1n} + W_2) \cdot (\langle S_z \rangle - S_0). \qquad \text{S3}$$

Assuming steady state conditions with respect to $\langle I_z \rangle$ ($\frac{d\langle I_z \rangle}{dt} = 0$ under continuous wave microwave irradiation at the EPR transition) and rearranging, an expression for the enhancement ε can be obtained:[2]

$$\varepsilon = \frac{\langle I_z \rangle}{I_0} = 1 + \frac{W_2 - W_0}{W_0 + 2W_{1e} + W_2} \cdot \frac{W_0 + 2W_{1\mathrm{e}} + W_2}{W_0 + 2W_{1\mathrm{e}} + W_2 + W_{1\mathrm{n}}} \cdot \frac{(\langle S_z \rangle - S_0)}{S_0} \cdot \frac{S_0}{I_0} \qquad \text{S4}$$
$$= 1 - \xi f s \frac{|\gamma_e|}{\gamma_n}$$

where ξ is the coupling factor, f is the leakage factor, s is the saturation factor and γ_e and γ_n are the electron and nuclear gyromagnetic ratios. The reader is referred to the main text for a discussion of these factors in the context of graphite anodes.

2 Electrochemistry of the different graphite stages

Three different lithiation stages of the Hitachi graphite anodes were measured under DNP conditions: dense stages 1 and 2 and dilute stage 2L. These stages were isolated electrochemically by cycling graphite anodes against Li metal in half cells. In all cases, two formation cycles (charge/discharge) were performed prior to the final lithiation to a lower cutoff voltage of 1 mV (stage 1), 76 mV (stage 2) and 120 mV (stage 2L) *vs* Li/Li^+ (Figure S2).

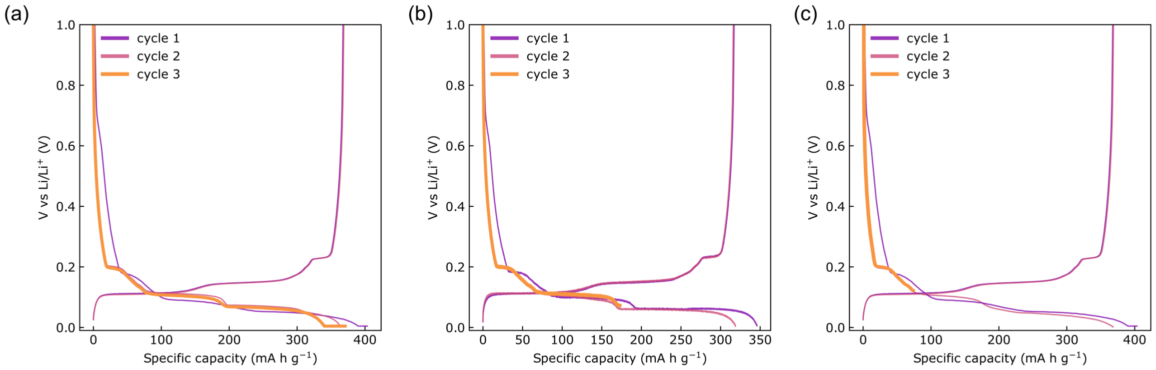

Figure S2. Electrochemical charge/discharge curves (charge = lithiation here) of Li:graphite half cells cycled with LP57 electrolyte at a charge rate of C/17 (assuming a theoretical capacity of 360 mAh g^{-1} (current density of 18.4 mAh). The cells were cycled for 2 cycles prior to be lithiated to lower cutoff voltage of 1 mV, 76 mV or 120 mV *vs* Li/Li^+ corresponding to stages 1 (a), 2 (b) and 2L (c), respectively. The thickness of the voltage curve for the final lithiation (cycle 3) is larger for ease of reading.

3 DNP ON/OFF spectra of the different stages at various temperatures

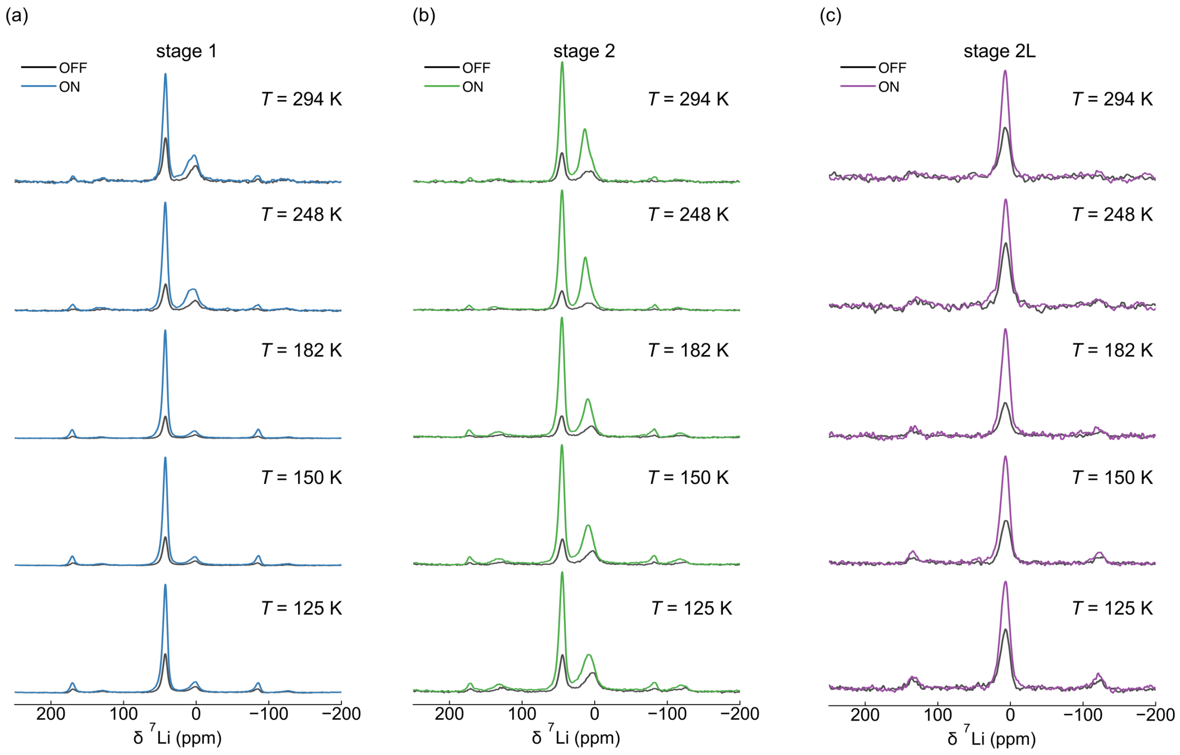

Figure S3. VT ^{7}Li DNP spectra of the stages 1 (a), 2 (b) and 2L (c) whose enhancement *vs* sample temperature was reported in Figure 5 in the main text. These spectra were recorded as Hahn echos at 20 kHz and using a klystron microwave source (5.2 W). The temperatures are quoted as sample temperatures as measured *ex situ* using the *T*-dependence of the T_1 relaxation of ^{79}Br in KBr for $T < 240$ K and the *T*-dependence of the ^{79}Br shift in KBr at higher temperatures. The recycle delays used were 10 s for $T < 240$ K, 5 s for $T > 240$ K. The samples were all Hitachi graphites diluted with 95 %wt quartz powder.

4 ^{7}Li enhancement using a 3.2mm probe

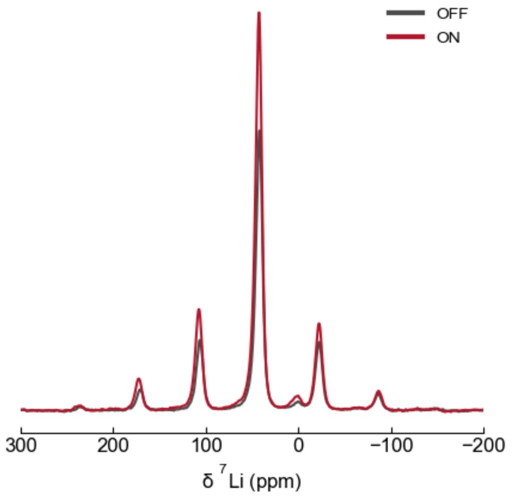

Figure S4. ^{7}Li ON/OFF spectra of stage 1 Hitachi graphite measured in a 3.2 mm DNP probe at 100 K, using a klystron microwave source (5.2 W). The MAS rate was 10 kHz and a recycle delay of 15 s was used. The sample was diluted here with 95 %wt KBr and packed in a 3.2 mm sapphire rotor. The observed enhancement factor was ~1.5.

5 Enhancement at different spinning speeds

The effect of the spinning speed on the observed enhancement was investigated on a stage 1 Hitachi graphite sample, diluted with 95 %wt quartz powder at room temperature and using a klystron microwave source. As discussed in the main text, at 30 kHz two main signals are observed: the bulk intercalated Li signal at 42.6 ppm and the SEI signal around ~0 ppm. At lower spinning speed an additional signal is observed in the microwave ON spectra at 11.6 ppm. The lower spinning speed spectra were recorded on the same rotor after the 30 kHz spectra were recorded. Due to experimental constraints, these experiments can only be run at room temperature, with no means to offset the increased sample temperature caused by frictional heating and microwave irradiation. In these conditions, it has been previously reported that Li ions can deintercalate graphite (ref), and this is indeed observed here, where the 11.6 ppm peak is assigned to a dilute stage environment, occurring on partial delithiation of the dense stages. It should be noted that this peak is only visible thanks to the OEDNP effect, which enhances dramatically this signal.

The LiC_6 signal at 42.6 ppm is enhanced by a factor of ~3.3 across all spinning speeds, indicating no strong dependence of the enhancement on the spinning speed.

However, the static signal recorded at the bottom of Figure S5 shows a substantial enhancement which allows to observe the quadrupolar transition of both the dense stage 1 and the newly formed dilute stage, with measured ν_Q ~ 15.5 kHz for the dense stage 1 and ~ 17.8 kHz for the dilute stage (close to the observed static ν_Q reported by Letellier *et al.*[10])

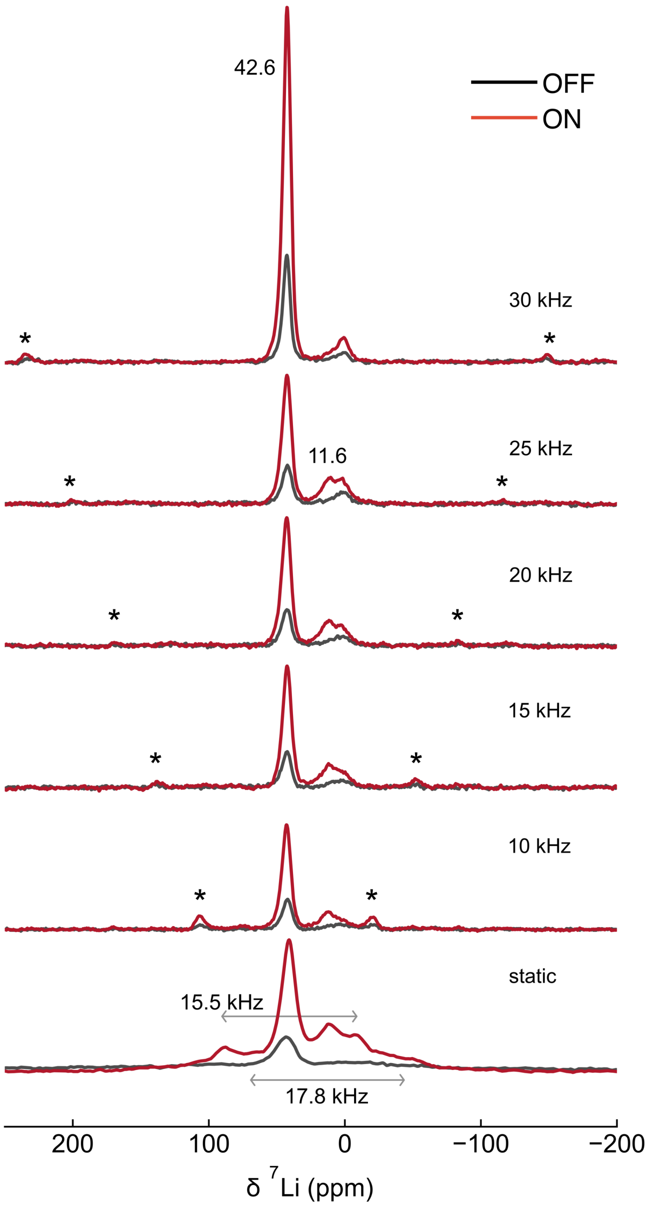

Figure S5. ^{7}Li ON/OFF Hahn echo spectra recorded for the same LiC_6 sample at different MAS rates, at room temperature, at 5.2 W microwave power and with a recycle delay of 5 s. The sample was stage 1 Hitachi graphite diluted with 95 %wt quartz powder.

6 DNP field profile for the lightly degraded stage 2 sample

Below is an example of the different in the enhancement maxima for the dense stage ^{7}Li peak compared to the dilute stage peak in a nominally stage 2 sample which underwent degradation during measurement. It can be seen that the enhancement maximum shifts to lower field (higher *g*-factor) for the dilute stage peak, consistent with our EPR experiments.

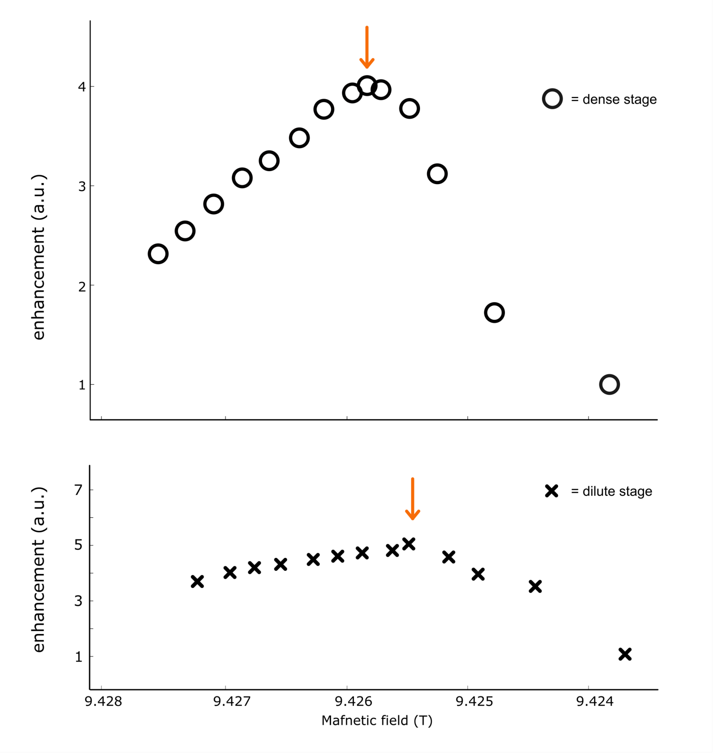

Figure S6. Field profile of the lightly degraded stage 2 sample (Hitachi), showing the dense stage peak (circles, top) and the dilute stage peak (crosses, bottom) satisfy the OEDNP matching condition at slightly different fields (maxima indicated by orange arrow), consistent with the different *g*-factors seen by EPR for dense and dilute stages. The spectra were recorded at 250 K (sample temperature), at 5.2 W, spinning at 20 kHz and with a recycle delay of 5 s, The sample was Hitachi graphite, nominally stage 2, diluted with 95 %wt quartz.

7 Natural abundance ^{6}Li DNP spectra

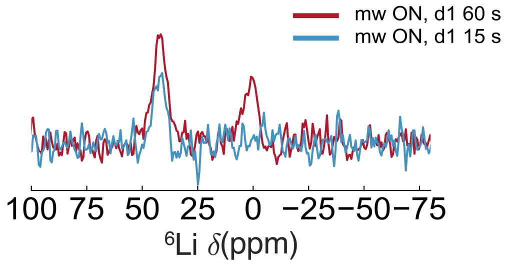

Figure S7. Natural abundance ^{6}Li DNP spectra (mw ON only) at different recycle delays (d1 = 15 s and 60 s) showing how longer delays are required to observe the diamagnetic signal.

8 ^{7}Li exsy spectra at variable mixing times

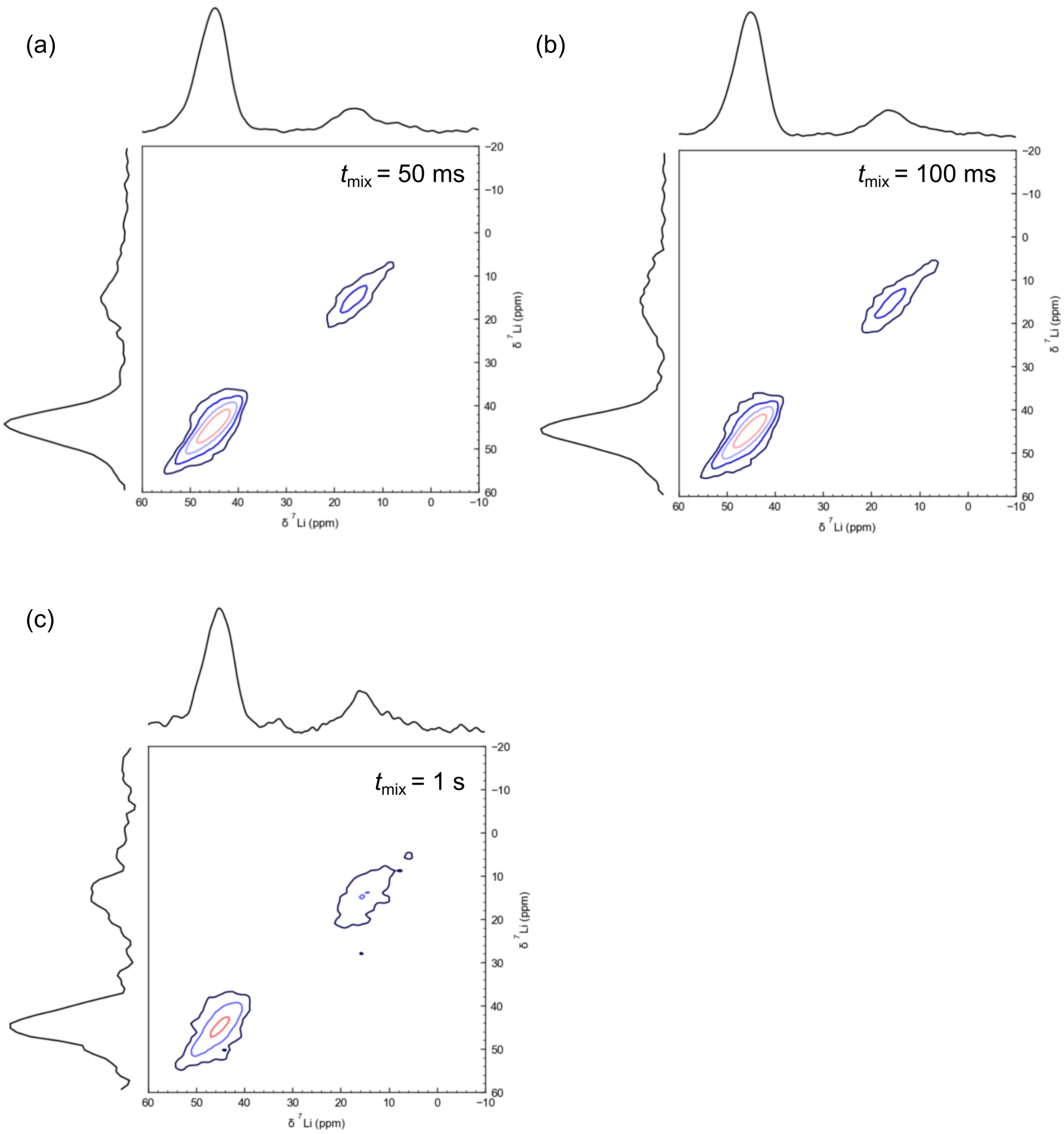

Figure S8. ^{7}Li-^{7}Li EXSY spectra of a stage 2 Hitachi graphite sample at variable mixing times: (a) 50 ms, (b) 100 ms, (c) 1s. The experiments were performed at room temperature, under microwave irradiation (5.2 W) and spinning at 20 kHz. A recycle delay of 5 s was applied throughout. No exchange peaks are observed, suggesting little chemical exchange or spin diffusion are present.

9 ^{1}H DNP field sweep

A ^{1}H DNP field profile was measured over a ~37 mT field range to look for any enhancement arising from any DNP mechanism, but the sweep produced no enhancement at any of the measured fields (see Figure S9 below). The sweep covered a broad enough region to satisfy the matching conditions for OE, solid effect and cross effect, these conditions based on the observation that the only EPR signal seen in these materials is centred around 2.003 at high field. In the Overhauser mechanism, irradiation at the EPR transition will produce signal enhancement (provided conditions are met, cf main text), regardless of the nucleus investigated, so the field should be the same for ^{7}Li, ^{6}Li, ^{13}C, ^{1}H etc. There is a possibility that solid effect might play a role, in this case not coming from conduction electrons but rather from localised defect spins at the surface. Their *g*-factor would also be expected to be around ~2 (as there would be similar and therefore negligible spin-orbit coupling). If they were localised, not concentrated (*i.e.* the electron-electron dipolar interaction is weak) and near protons, the solid effect would arise when irradiating the ZQ/DQ transitions, seeing positive and negative enhancement maxima separated by the ^{1}H Larmor frequency. At 9.4 T, 400 MHz corresponds to ~14 mT, which were abundantly covered in this sweep.

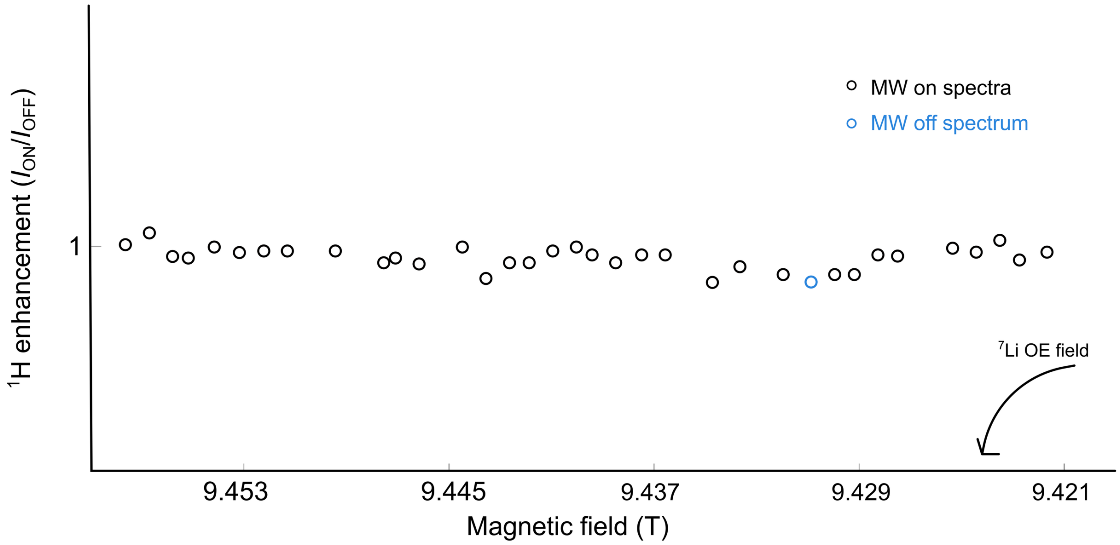

Figure S9. DNP field sweep of a LiC_{12} sample tracking the ^{1}H signal at different fields. No enhancement was observed at any stage. The measurements were conducted at 100 K, using the 1.3 mm probe, spinning at 25 kHz, as Hahn echo experiments with a recycle delay of 8 s and using 5.2 W microwave power (klystron).
